# Thermodynamics and Chemical Behavior of Uranyl Superoxide at Elevated Temperatures

**DOI:** 10.1021/acsmaterialsau.1c00033

**Published:** 2021-08-26

**Authors:** Dmytro V. Kravchuk, Tori Z. Forbes

**Affiliations:** Department of Chemistry, University of Iowa, Iowa City, Iowa 52242, United States

**Keywords:** Uranyl superoxide, Uranium, Superoxide, Carbon capture

## Abstract

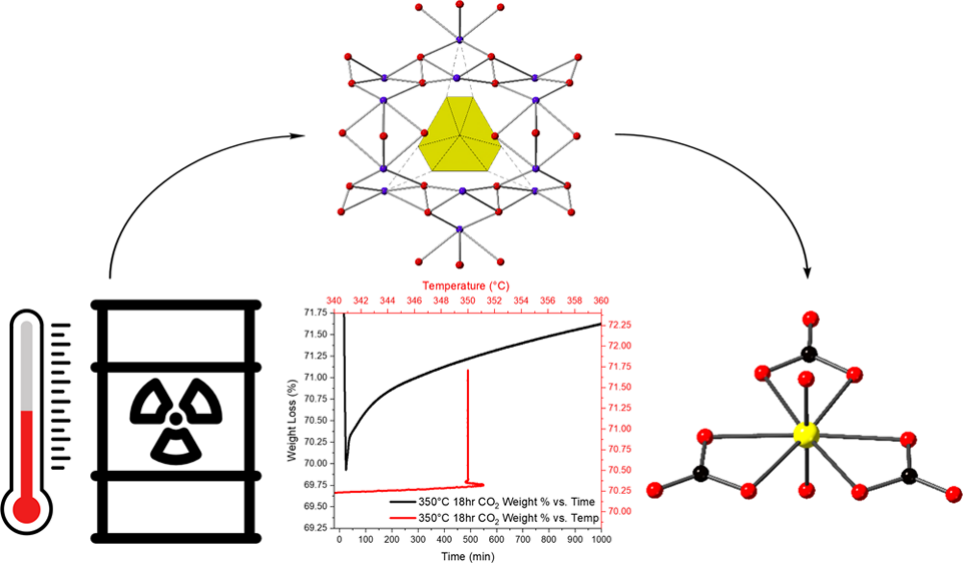

Understanding the alteration mechanisms of UO_2_-based nuclear fuel has a range of practical implications for both short- and long-term storage of spent fuel rods and environmental ramifications for the mobility of radioactive material at the Chernobyl and Fukushima sites. The major identified alteration phases on the surface of nuclear waste are analogues of schoepite UO_3_·2H_2_O, studtite UO_2_(O_2_)·4H_2_O, rutherfordine UO_2_CO_3_, and čejkaite Na_4_UO_2_(CO_3_)_3_. While α-radiolysis has been shown to cause the ingrowth of uranyl peroxide alteration phases, the prevalence of uranyl carbonate phases on solid waste forms has not been mechanistically explained to date, especially since the alteration chemistry is largely affected by the high temperatures of the spent nuclear material. Herein, we demonstrate the first mechanistic link between the formation of the uranyl superoxide (**KUPS-1**) phase, its reactivity at temperature ranges relevant to the spent nuclear fuel (40–350 °C), and its thermodynamic transformation into a potassium uranyl carbonate mineral phase, agricolaite K_4_[UO_2_(CO_3_)_3_], using thermogravimetric analysis, calorimetry, vibrational spectroscopy, and powder X-ray diffraction techniques. The thermodynamics data reveal the metastability of the uranyl superoxide **KUPS-1** phase through decomposition of the hydrogen peroxide within the solid-state lattice. Increasing the temperature does not result in the breakdown of the superoxide anion bound to the uranyl cation but instead enhances its reactivity in the presence of CO_2_ gas, resulting in potassium carbonate phases at intermediate temperatures (150 °C) and in uranyl carbonate phases at higher temperatures (350 °C).

## 1 Introduction

The successful containment of nuclear material in cooling ponds, dry casks, geological repositories, and accident sites requires an understanding of the surface chemistry and potential alteration of U^IV^O_2_ fuel pellets.^1,2^ Complex solute matrices with varying conditions (humidity levels, exposure to water, elevated temperatures, high radiation fields, etc.) all factor into the chemistry occurring on the surface of U^IV^O_2_-based nuclear waste and result in a vast array of U^VI^ corrosion phases.^3,4^ For example, detailed SEM and X-ray diffraction studies of the Chernobyl corium identified studtite UO_2_(O_2_)·4H_2_O, metastudtite UO_2_(O_2_)·2H_2_O, rutherfordine UO_2_CO_3_, and čejkaite Na_4_UO_2_(CO_3_)_3_ as the main alteration phases on the surface of the Chernobyl corium exposed to the atmosphere inside the sarcophagus.^5−8^ Even Chernobyl lava samples collected in 1990 inside the “Sarcophagus” and stored under ambient laboratory conditions for 20 years showed clear signs of chemical alteration.^9^ Similarly, metaschoepite [(UO_2_)_8_O_2_(OH)_12_](H_2_O)_10_, studtite, and metastudtite have been observed on surfaces of spent nuclear fuel rods in water cooling ponds.^10^ Damaged reactor cores at the Fukushima nuclear power plant are expected to form uranyl peroxide nanoclusters that are thermodynamically stable intermediate products of uranium oxide nuclear fuel being exposed to seawater.^11^ Such uranyl peroxide nanoclusters, if they are sufficiently concentrated, might precipitate and convert to alkali uranyl carbonates, oxyhydroxides, silicates, and other solid-state compounds. Moving forward, the proposed geological repository sites, such as Yucca Mountain, NV, may have the right chemical conditions to further propagate the formation of corrosion products, which can affect the mobility and transport of radionuclides within the subsurface environment.^12,13^

Our current mechanistic understanding of the alteration chemistry of U^IV^ nuclear waste begins with the interaction of ionizing radiation with water molecules (either by adsorption from the atmosphere or by direct contact with natural waters) at the surface of radioactive fuel rods.^14,15^ The ionizing radiation interacts with water molecules at the solid–liquid or solid–gas interface, resulting in either the absorption event generating excited water molecules H_2_O* or the ionization event emitting the subexcitation electron e^–^ and ionized water H_2_O^•+^.^16,17^ The excited water molecules undergo homolytic cleavage of the O–H bond, yielding hydroxy OH^•^ and hydrogen H^•^ radicals. At this stage of radiolysis, the reactive species (e^–^_aq_, H_2_O^•+^, OH^•^, H^•^) can react with each other, the surrounding water molecules, or dissolved O_2_ gas within the solution, resulting in a variety of products, such as superoxide radical O_2_^•–^, hydroperoxyl radical OOH^•^, peroxide anion O_2_^2–^, hydroperoxyl anion OOH^–^, and molecular hydrogen peroxide H_2_O_2_ (the speciation is highly dependent on the pH of the solution). The major product of the radiolysis is H_2_O_2_, resulting from the direct combination of two hydroxyl radicals OH^•^. The amount of dissolved molecular O_2_ within water also plays a significant role in the amounts of H_2_O_2_ and O_2_^•–^/OOH^•^ produced on the basis of irradiation studies of aerated solutions in comparison to inert gas purged solutions.^18^ It was found that the concentrations of H_2_O_2_ and O_2_^•–^/OOH^•^ produced upon irradiation of aerated solutions was about 10^4^ times higher in comparison to inert gas analogues due to the reactivity of e^–^_aq_ and OH^•^ toward molecular oxygen.^19^ Reactive oxygen species (e.g., H_2_O_2_) can interact with the surface of nuclear waste, oxidizing U^IV^ to the uranyl cation (U^VI^O_2_^2+^), which opens up a major pathway to the formation of uranyl peroxide alteration phases—studtite ([UO_2_(O_2_)(H_2_O)_2_]·2H_2_O) and metastudtite ([UO_2_(O_2_)(H_2_O)_2_]).^10,20−23^ Both mineral phases have been shown to solubilize at slightly basic pH values to form uranyl peroxide closed-cage clusters and have the potential to mobilizing U^VI^ into the surrounding aqueous environment.^24,25^ Additionally, upon the γ-irradiation of suspensions containing studtite and metastudtite in the presence of HCO_3_^–^ anions, the rapid dissolution of both minerals was observe,d resulting in uranyl peroxo-carbonate compounds.^26^

The formation of hydrogen peroxide in aqueous solution relies on the reactivity of reactive intermediates, such as the recombination of hydroxyl OH^•^ radicals in a major pathway, as well as hydrogen radical H^•^ capture by OOH^•^ in a minor pathway, and there is initial evidence that the OOH^•^ species may bind and stabilize through interactions with the U^VI^O_2_^2+^ cation. We have recently shown that O_2_^•–^ and OOH^•^ binds to the uranyl cation, resulting in the isolation of the metastable uranyl superoxide phase **KUPS-1**, K_4_[UO_2_(O_2_^2–^)_2_(O_2_^–^)_0.5_(OOH^•^)_0.5_](OOH^–^)_1.5_(H_2_O_2_)_2.5_(H_2_O)_4_ (due to proton transfer within the framework, the molecular formula could also be rewritten as K_4_[UO_2_(O_2_^2–^)_2_(O_2_^–^)](OOH^–^)(H_2_O_2_)_3_(H_2_O)_4_).^27,28^ Typically, these dioxygen radical species are not stable and their half-life is approximately ∼0.1 ms at concentrations of 10^–4^ M.^29^ The solid uranyl superoxide can be stabilized for approximately 12 h in the mother liquor and for up to 1 week in a vacuum desiccator. This suggests that it is possible to stabilize a transient uranyl superoxide species on the surface of enriched UO_2_ fuels, and *in situ* Raman experiments performed by Canizarès et al. provided some initial evidence that this may occur within irradiated UO_2_ samples.^14^

Our previous work on the uranyl superoxide phase **KUPS-1** also demonstrated that it will react with CO_2_ in the air and may help explain the prevalence of uranyl carbonate phases on the surface of UO_2_ fuels.^5^ The chemistry of uranyl superoxide upon exposure to CO_2_ from the atmosphere led to the formation of mixed uranyl carbonate phases K_4_[UO_2_(CO_3_)_2_(O_2_)]·H_2_O and K_3_Na[UO_2_(CO_3_)_3_]·H_2_O (grimselite) both in solution and in the open air,^30^ and such reactivity has direct effects on our understanding of corrosion pathways of UO_2_ fuels. We hypothesize that uranyl carbonate alteration phases can be produced from the reaction between molecular CO_2_ in the air and the superoxide anion that is continuously produced from α-radiolysis occurring at the solid–gas and solid–liquid interfaces. To continue to test this hypothesis, we need to consider the effect of environmental conditions associated with the surface of UO_2_ -based spent nuclear fuel.

While our initial findings confirmed the link between the reactive oxygen species and the formation of uranyl carbonate phases, assessing the potential of superoxide anion in the alteration of spent nuclear fuel should take into account the thermal load associated with the high radiation field.^31−33^ When spent nuclear fuel is placed in either a nuclear fuel pool or dry casket storage, the temperature of the fuel element ranges from 175 to 410 °C.^34,35^ Thermodynamic studies of studtite and metastudtite by Guo et al. and Rey et al. indicated that decomposition occurs under these relevant temperature regimes to form amorphous U_2_O_7_ and UO_3+*x*_ materials, which can release significant amounts of molecular oxygen and have the potential to cause overpressurization of storage vessels for yellow cake.^36,37^ Similarly, an evaluation of the thermal and chemical behavior of the uranyl superoxide **KUPS-1** is needed to assess the relevance of the reactive oxygen species in the process of alteration of spent nuclear fuel at elevated temperatures.

Herein, we report the thermal stability and reactivity of the solid-state compound **KUPS-1** at elevated temperatures (40–350 °C) for up to 18 h. The transformations and energetics of the **KUPS-1** compound are monitored using thermogravimetric analysis combined with differential scanning calorimetry. The chemical behavior of uranyl superoxide is assessed on the basis of vibrational (infrared and Raman) spectroscopy and powder X-ray diffraction at varying temperatures (40, 150, and 350 °C) and environments (air, N_2_, and CO_2_). In addition, we explore the differences in thermal behavior of the **KUPS-1** compound in comparison to the reactive U_2_O_7_ phase formed from the dehydration of studtite to determine the relationships between these two materials.

## 2 Experimental Methods

### 2.1 Materials and Synthesis

All aqueous solutions were prepared using Millipore water (18.2 MΩ), and the chemicals purchased were used directly without further purification. *Caution! UO*_2_*(NO*_3_*)*_2_*·6H*_2_*O contains radioactive*^238^*U, which is an α emitter and like all radioactive materials must be handled with care. These experiments were conducted by trained personnel in a licensed research facility with special precautions taken toward the handling, monitoring, and disposal of radioactive materials*. The compound **KUPS-1** was synthesized according to a previously published procedure.^27^ The general synthetic procedure included combining 0.2 mL of a 0.05 M uranyl nitrate hexahydrate (International Bio-Analytical Industries Inc., 99.99%) stock solution in methanol with 0.15 mL of pure methanol (Fisher Chemical, ACS 99.9%) and 2.5 mL of pure benzyl alcohol (Alfa Aesar, 99%) in a 20 mL scintillation vial. A separate aqueous layer composed of 0.2 mL of a 30% aqueous solution of H_2_O_2_ (Fisher Chemical, ACS 99.9%), 0.3 mL of 0.05 M aqueous solution of *N*-(phosphonomethyl)iminodiacetic acid hydrate (Sigma-Aldrich, 95%) with sodium hydroxide (added for solubility), and 0.2 mL of 1.0 M aqueous potassium hydroxide (Sigma-Aldrich, 90%) solution was gently layered on top of the organic layer. The scintillation vial was capped and stored in the dark. Bright yellow, blocky crystals (300 μm) appeared on the bottom and the walls of the vials overnight in a yield of 54% based on U. These synthetic conditions represent the optimized yields, and the formation of **KUPS-1** is highly reproducible.

On average 40–50 synthetic vials were prepared at a time to yield enough solid **KUPS-1** material for the experiments described herein. Each experimental vial was vortexed and agitated to suspend the solid **KUPS-1** material in the solution. The suspension was quantitatively transferred into a 15 mL plastic conical tube and centrifuged at 6000 rpm for 5–10 min. Each conical tube was carefully decanted, and 10 mL of methylene chloride (Fisher Chemical, >99.5%) was added to the **KUPS-1** sediment. The resulting suspension was further centrifuged at 6000 rpm for 5 min, which forced any excess water trapped within the **KUPS-1** powder into the top aqueous layer. The liquids were decanted, and the **KUPS-1** solid was transferred onto a watch glass that was then placed in a vacuum desiccator to dry for 30 min. For the long-term storage of **KUPS-1**, the solid was transferred into a 20 mL scintillation vial, which was then connected to a Schlenk line. The vial containing **KUPS-1** was evacuated and back-filled with argon gas (Praxair, 99.98%) three times, covered with Parafilm, and placed in the freezer (−20 °C) for storage due to the air and temperature sensitivity of the compound.

The synthesis of studtite was performed according to modified literature procedures.^25,38^ An initial solution containing 10 mL of a 0.2 M aqueous solution of uranyl nitrate hexahydrate was treated with concentrated nitric acid until the pH was <1 while it was stirred with a magnetic stir bar. Following the acidification of the uranyl nitrate stock solution, 30% aqueous H_2_O_2_ was added dropwise at the rate of one drop per minute. Immediately after the addition of H_2_O_2_, a pale yellow-white precipitate (studtite) formed from the solution. Hydrogen peroxide was added dropwise until the pale yellow-white sediment stopped forming within the reaction vessel. The reaction mixture was transferred into a 15 mL plastic conical tube and centrifuged at 6000 rpm for 10 min. The suspension was decanted, and the light yellow precipitate was transferred onto a watch glass to dry in the vacuum desiccator overnight. Raman spectroscopy and PXRD analysis confirmed the identity and purity of the resulting studtite material.

### 2.2 Thermal Analysis

Thermogravimetric analysis was performed on a TA Instruments Q500 thermogravimetric analyzer. The sample chamber was purged using either nitrogen (Praxair 99.98%) or carbon dioxide gas (Praxair 99.5%), depending on the specific experiment. Approximately 15–20 mg of the powdered **KUPS-1** sample was placed on a preweighed aluminum pan and heated from 25 to 550 °C with at a rate of 5 °C min^–1^. For isothermal experiments, the samples were heated from 25 °C to three target temperatures, 40, 150, and 350 °C, at a rate of 5 °C min^–1^. When a target temperature was reached, the sample was held isothermally for 18 h under a nitrogen or carbon dioxide atmosphere with continuous monitoring for weight changes.

Differential scanning calorimetry measurements were performed using a TA Instruments Q100 differential scanning calorimeter. Samples were prepared by placing 2.5–7.5 mg of pristine **KUPS-1** powder on an aluminum sample pan, which was capped with an aluminum lid and hermetically sealed with an encapsulating press. An empty hermetically sealed aluminum pan was used during each measurement as a reference. The sample chamber was purged with a 1:1 mixture of ultrapure helium (Praxair 99.998%) and ultrapure nitrogen (Praxair 99.998%) at a flow rate of 20 mL min^–1^. All samples were allowed to equilibrate at 25 °C at the start of the experiment, followed by a temperature ramp at the rate of 5 °C min^–1^ until the temperature reached 350 °C. Measurements were replicated three times on independent samples to ensure reproducible results. The system was calibrated with an indium pellet calibration standard (99.999%), resulting in *K*_cell_ = 1.14450. TA Instruments TRIOS software was used to determine the peak temperature and enthalpy of each thermal event.

### 2.3 Vibrational Spectroscopy

Solid-state Raman spectra were acquired on a SnRI high-resolution Sierra 2.0 Raman spectrometer equipped with 785 nm laser energy and a 2048 pixel TE-cooled CCD. The laser power was set to the maximum output value of 15 mW, giving the highest achievable spectral resolution of 2 cm^–1^. Each sample was irradiated for an integration time of 5–30 s (depending on the sample) and automatically reiterated three times in multiacquisition mode. Three Raman spectra were acquired per sample, averaged together, and normalized on the basis of the laser power and integration time. FT-IR spectra were collected on a Nicolet Nexus FT-IR spectrometer from 500 to 4000 cm^–1^. Approximately 10–20 mg of the fine **KUPS-1** powder was mixed with an anhydrous KBr salt and pressed into a transparent pellet for data collection. To accurately process the vibrational signals observed, the background was subtracted, multiple peaks were fit using a peak analysis protocol with Gaussian and Lorentz functions, and all the fitting parameters were converged in the OriginPro 9.1.0 (OriginLab, Northampton, MA) 64-bit software.

### 2.4 Powder X-ray Diffraction

Powder X-ray diffraction data were collected on a Bruker D8 Advance diffractometer with nickel-filtered Cu Kα radiation (λ = 1.5418 Å), voltage 40 kV and current 40 mA, in the continuous mode with a scan range of 5–60° 2θ and a step size of 0.05°. Samples were ground to fine powders using a mortar and pestle and run using zero-background silica sample holders. Processing of the PXRD patterns, background subtraction, smoothing, Kα_2_ stripping, and peak selection were done using PreDICT indexing software from ICDD. The PXRD diffractograms were matched using the ICDD PDF-4+ database.

## 3 Results and Discussion

**Figure 1:**
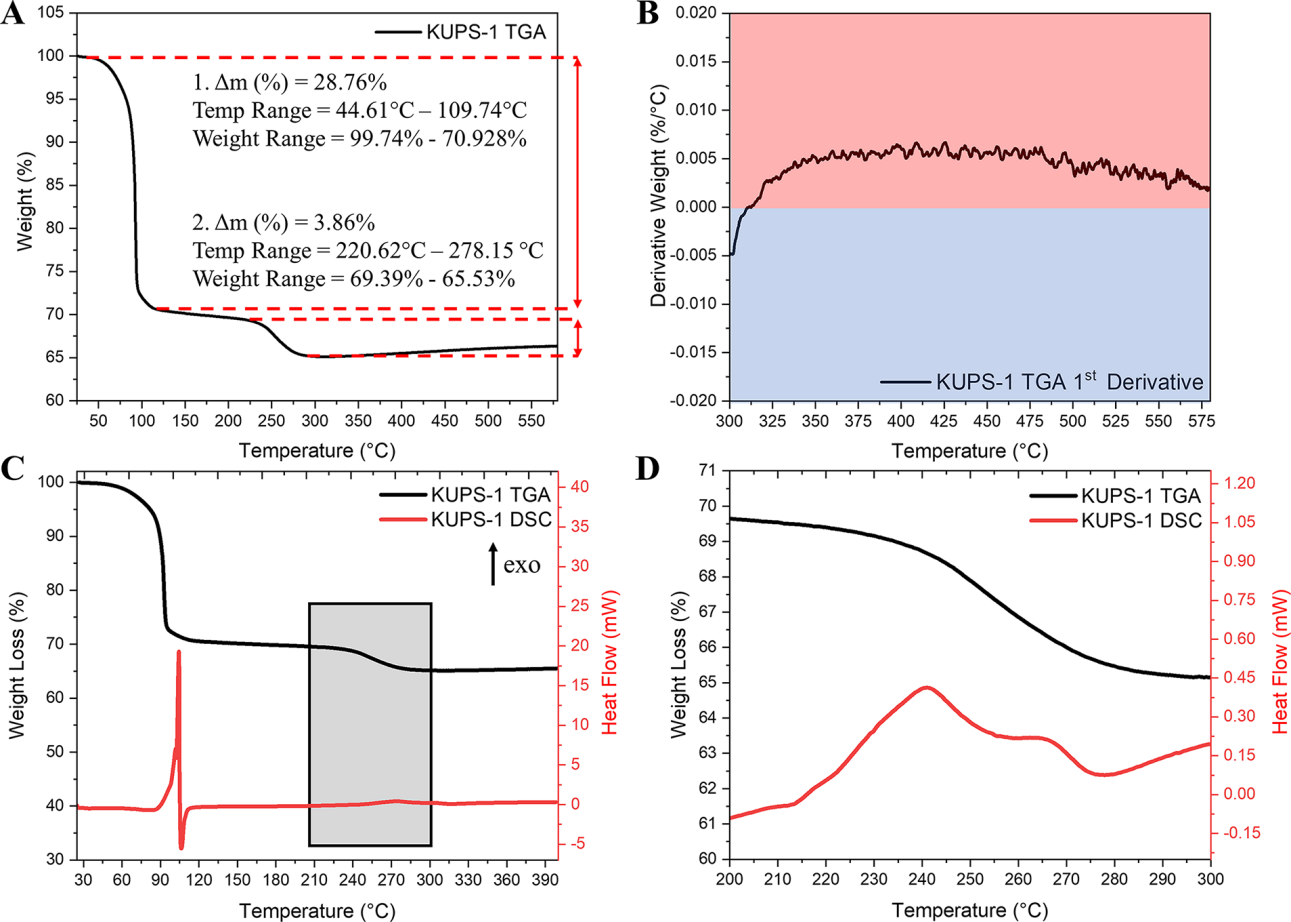
(A) TGA curve of **KUPS-1** containing two weight loss steps at 90 and 260 °C that are highlighted with red dashed lines. (B) Derivative weight versus temperature of the TGA curve for **KUPS-1** demonstrating a positive slope (red background) in the region from 315 to 580 °C. A blue background isused for negative slope values. (C) Overlaid TGA (black) and DSC (red) graphs matching the weight loss to the energetic transitions. The gray box indicates the second energetic transition. (D) Enlarged TGA/DSC curve in the temperature range 200–300 °C providing additional visualization of the second exothermic transition for the **KUPS-1** material.

Bulk crystalline **KUPS-1** was first evaluated with TGA to assess the thermal stability and resulting weight loss. The TGA curve of **KUPS-1** revealed a stepwise decomposition of **KUPS-1** with two major weight loss steps associated with (i) a loss of three hydrogen peroxide molecules, one hydroperoxyl anion, and four water molecules (experimental weight loss 28.76%, theoretical weight loss 28.43%, onset temperature 44.61 °C, end temperature 109.74 °C) and (ii) a subsequent loss of molecular oxygen (net weight loss 3.86%, theoretical weight loss 4.37%, onset temperature 220.62 °C, end temperature 278.15 °C) (Figure 1A). On the basis of the weight loss, the decomposition reaction should follow K_4_[UO_2_(O_2_^2–^)_2_(O_2_^–^)](OOH^–^)(H_2_O_2_)_3_(H_2_O)_4_ → K_4_UO_8_ → K_4_UO_6_. However, the TGA curve at temperatures of 300 °C and above demonstrates a noticeable weight gain, as supported by the positive values (≥0) of the derivative of the **KUPS-1** TGA curve (Figure 1B). We hypothesize that the intermediate phases K_4_UO_8_ and K_4_UO_6_ bearing the superoxide anion can react with trace levels of CO_2_ in the sample chamber, resulting in a small amount of potassium carbonate, uranyl carbonate, and uranyl oxide contaminants. This may account for the differences in the predicted and experimental weight losses that occur for the second step. The reactivity of the material at different temperatures will be explored in detail (*vide infra*).

DSC measurements indicate three concurrent events in the temperature region from 75 to 110 °C and a second set of exothermic peaks at 210–280 °C (Figure 1C). Within the lower temperature region, the there are two highly exothermic peaks and a third endothermic reaction, which is aligned with the initial weight loss of three hydrogen peroxide molecules, one hydroperoxyl anion, and four water molecules. The overall enthalpy of the transition was exothermal and was calculated to be −148.78 ± 4.27 kJ/mol. We believe that the exothermic peaks correspond to the breakdown of the H_2_O_2_ and OOH^–^ molecules and that the endothermic peak corresponds to the removal of 4 mol of H_2_O.

While we do not have the exact enthalpies associated with these steps, we can utilize values associated with similar materials and processes to provide an estimate to evaluate the accuracy of the DSC measurements. First, if we consider the enthalpy of decomposition of H_2_O_2_ to occur via disproportionation, then we can use the value of −196.1 kJ/mol for that process.^39^ Second, we need to consider the dehydration step. Literature values are available for the dehydration enthalpy for interstitial water groups within uranyl nitrate hexahydrate and range between +52.43 and +61.39 kJ/mol.^40^ From these values, we can then estimate the overall decomposition of the first step for **KUPS-1** to range between −182.48 and −146.64 kJ/mol. Thus, our experimental value from the DSC measurement occurs within the predicted range.

Interestingly, the second weight loss in the temperature range 210–280 °C corresponding to a loss of molecular oxygen is also associated with three exothermic reactions (Figure 1D), and the enthalpy for this region was measured as −29.04 ± 1.55 kJ/mol. The two consecutive exothermic processes during **KUPS-1** decomposition are vastly different from the decomposition energetics of studtite, where weight losses at 90 and 240 °C both correspond to endothermic reactions.^36^ The first endothermic step for studtite corresponds to the dehydration of the interstitial water groups to form metastudtite, and the second corresponds to an additional removal of the ligated water and the formation of an amorphous intermediate state. An exothermic transition has been noted by Guo et al., where the material undergoes a transition at 550 °C from the amorphous state to form the α-UO_2.9_ phase. This suggests that the second exothermic step in the DSC of **KUPS-1** may also be related to a phase transition for the material and not dehydration of the material.

**Figure 2:**
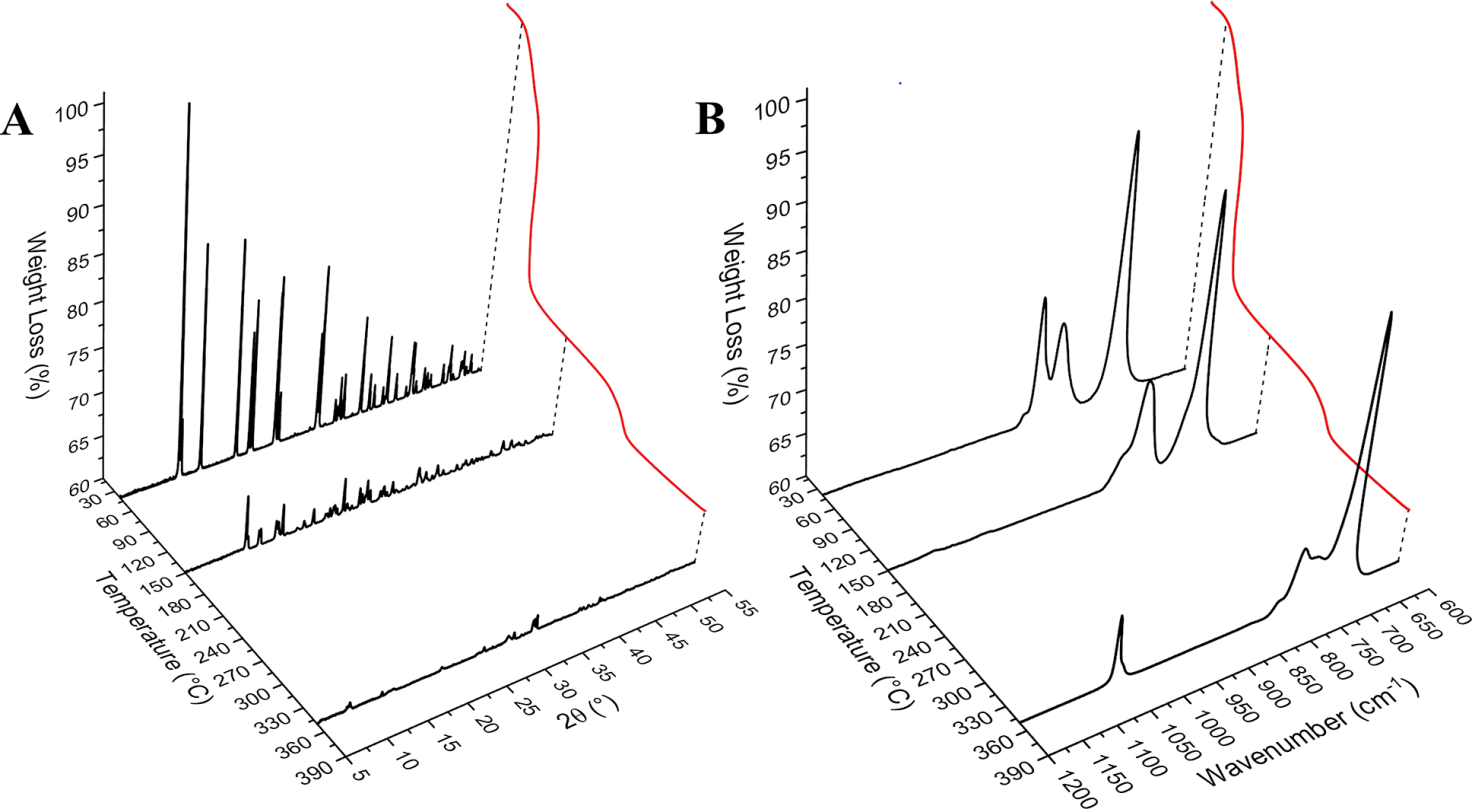
(A) Waterfall plots of PXRD patterns of **KUPS-1** taken at 40, 150, and 350 °C and correlated to the TGA curve (red). (B) Waterfall plots of the Raman spectra of KUPS-1 taken at 40, 150, and 350 °C and correlated to the TGA curve (red).

To better assess the decomposition pathway of **KUPS-1** and identify the intermediate and final products, we obtained PXRD patterns and Raman spectra of the quenched samples at 40, 150, and 350 °C, as shown in Figure 2. The PXRD pattern of **KUPS-1** at 40 °C matched the pattern calculated from SCXRD data for **KUPS-1**, indicating no major changes to the material at the start of the heating process. Increasing the temperature of the sample to 150 °C results in a significant decrease in the crystallinity of the sample (Figure 2A). The diffraction pattern is challenging to match, but major reflections associated with **KUPS-1** can still be observed alongside additional peaks that suggest the presence of a secondary decomposition phase. A reduced intensity of the reflections is also observed in the sample quenched at 350 °C, where the sample is mostly amorphous with a mixture of poorly crystalline material that could not be clearly identified using PXRD.

Raman spectra of associated with the **KUPS-1** thermal samples did provide some insight into the transformation from significant changes in the vibrational bands in the analyzed samples (detailed Raman spectra fittings are available in the Supporting Information). The Raman spectrum of **KUPS-1** at 40 °C showed the expected vibrational bands for the pristine **KUPS-1** material in the spectroscopic window of interest. The high-intensity band at 728 cm^–1^ was assigned to the ν_1_ symmetric stretch of uranyl. Bands occurring from 806 to 851 cm^–1^ aligned with ν_1–3_ motions of the peroxide coordinated to the uranyl,^41^ while modes at 860 cm^–1^ aligned with ν_O–O_ stretching of OOH^–^/H_2_O_2_ species coordinated to the hydrated potassium network.^42^ The mode at 882 cm^–1^ has previously been assigned to the ν_O–O_ stretching of O_2_^•–^/OOH^•^. When the **KUPS-1** sample was heated to 150 °C, the feature associated with the uranyl ν_1_ symmetric stretch broadened from the centroid at 728 cm^–1^ and was fit to three bands at 684, 712, and 735 cm^–1^. Such Raman shifts of the uranyl cation do not suggest a complete change in the uranyl speciation but rather a change in the intermolecular interactions with the uranyl-oxo groups. This is mostly likely associated with the disturbance of the H-bonding network due to the loss of water and hydrogen peroxide molecules at 150 °C. Additional vibrational modes are observed at 793, 805, and 835 cm^–1^. In addition, the bands at 860 cm^–1^ (ν_O–O_ stretching of OOH^–^/H_2_O_2_) and 882 cm^–1^ (ν_O–O_ stretching of O_2_^•–^/OOH^•^) have disappeared from the spectra and support the removal of H_2_O_2_/OOH^–^ from the lattice.

Dembowski et al. noted splitting and activation of stretching modes for the alkali and alkali-earth uranyl triperoxide compounds, where the uranyl stretching bands were reported between 677 and 738 cm^–1^.^41^ Looking specifically at the K_4_[UO_2_(O_2_)_3_]·4H_2_O compound, they observed the ν_1_ UO_2_^2+^ mode at 715 cm^–1^ and computational analysis predicted the presence of the asymmetric stretching feature at 778 cm^–1^. In addition, spectral signals were observed at 814, 825, and 843 cm^–1^ that the corresponded to peroxide stretching modes. Dembowski et al. also noted bands near 680 cm^–1^ for the sodium uranyl triperoxide system and attribute these features to differences in the hydrogen bonding networks and uranyl-cation interactions. This previous work then supports our analysis for **KUPS-1** at 150 °C that assigns the three bands between 680 and 735 cm^–1^ to the uranyl symmetric stretch. In addition, it suggests that 793 cm^–1^ may be associated with an activated ν_3_ uranyl asymmetric stretch and the vibrational modes at 805 and 835 cm^–1^ correspond to the peroxide ligands bound to the uranyl.

The most dominant spectral feature associated with the **KUPS-1** sample quenched at 350 °C is located at 696 cm^–1^, along with a band at 775 cm^–1^. These bands match well with the previously reported values for the potassium uranate (K_2_UO_4_) phase.^43^ The presence of carbonate phases were confirmed by bands at 1052 and 1060 cm^–1^ corresponding to the ν_4_ stretch of the CO_3_^2–^ coordinated to the uranyl cation and to the potassium cation (K_2_CO_3_), respectively. This leaves the band at 806 cm^–1^, which is identical with the very strong band reported by Anderson et al. for K_4_[(UO_2_)(CO_3_)] that was assigned to the ν_1_ uranyl symmetric stretch for the uranyl tricarbonate anion.^44^ The mode at 746 cm^–1^ may be associated with the CO_3_^2–^ anion coordinated to the uranyl cation and is observed in a range of uranyl tricarbonate species. This leaves the band at 711 cm^–1^, which may indicate that some of the poorly crystalline dehydrated uranyl superoxide/peroxide phase remains in the solid phase at this temperature because a similar peak (along with a band at 806 cm^–1^) was also observed after heating to 150 °C.

Raman spectroscopy combined with a PXRD analysis of **KUPS-1** quenched samples revealed a thermal degradation of the hydrated potassium peroxide uranyl superoxide material at 40 °C to a poorly crystalline, dehydrated potassium uranyl superoxide/uranyl peroxide material at 150 °C. This was followed by a further transformation to a mixed phase of amorphous (potassium uranate, amorphous potassium uranyl superoxide/uranyl peroxide, and potassium carbonate) phases and poorly crystalline uranyl carbonate at 350 °C. Crystalline potassium uranates are achieved through high-temperature solid-state reactions (450–850 °C)^45^ or CO_3_/OH fluxes;^46−48^ thus, the amorphous nature of the material fits well with the PXRD results we observe through the thermal decomposition of **KUPS-1**. The ingrowth of uranyl and potassium carbonates at 350 °C suggests the continuous reactivity and stability of the superoxide anion coordinated to the uranyl even at elevated temperatures, resulting in carbon capture. It was also noted in the TGA of the **KUPS-1** material that a weight gain continued after 350 °C that may correspond to additional reactivity of the uranyl superoxide/peroxide phase at this temperature. Disproportionation of the superoxide with water vapor (2O_2_^•–^ + H_2_O → O_2_ + HOO^–^ + OH^–^) or simple reduction to O_2_ gas will result in either no mass difference or a loss, but a carbonation reaction results in a mass gain of 28 g/mol with the reaction following the addition of one CO_2_ molecule and loss of 1/2 O_2_ molecule. Hence, we monitored the weight gain of the **KUPS-1** material by performing an isothermal heating experiment within TGA at 40, 150, and 350 °C under both N_2_ and CO_2_ gas to evaluate what conditions are optimal for the carbon capture process.

**Figure 3:**
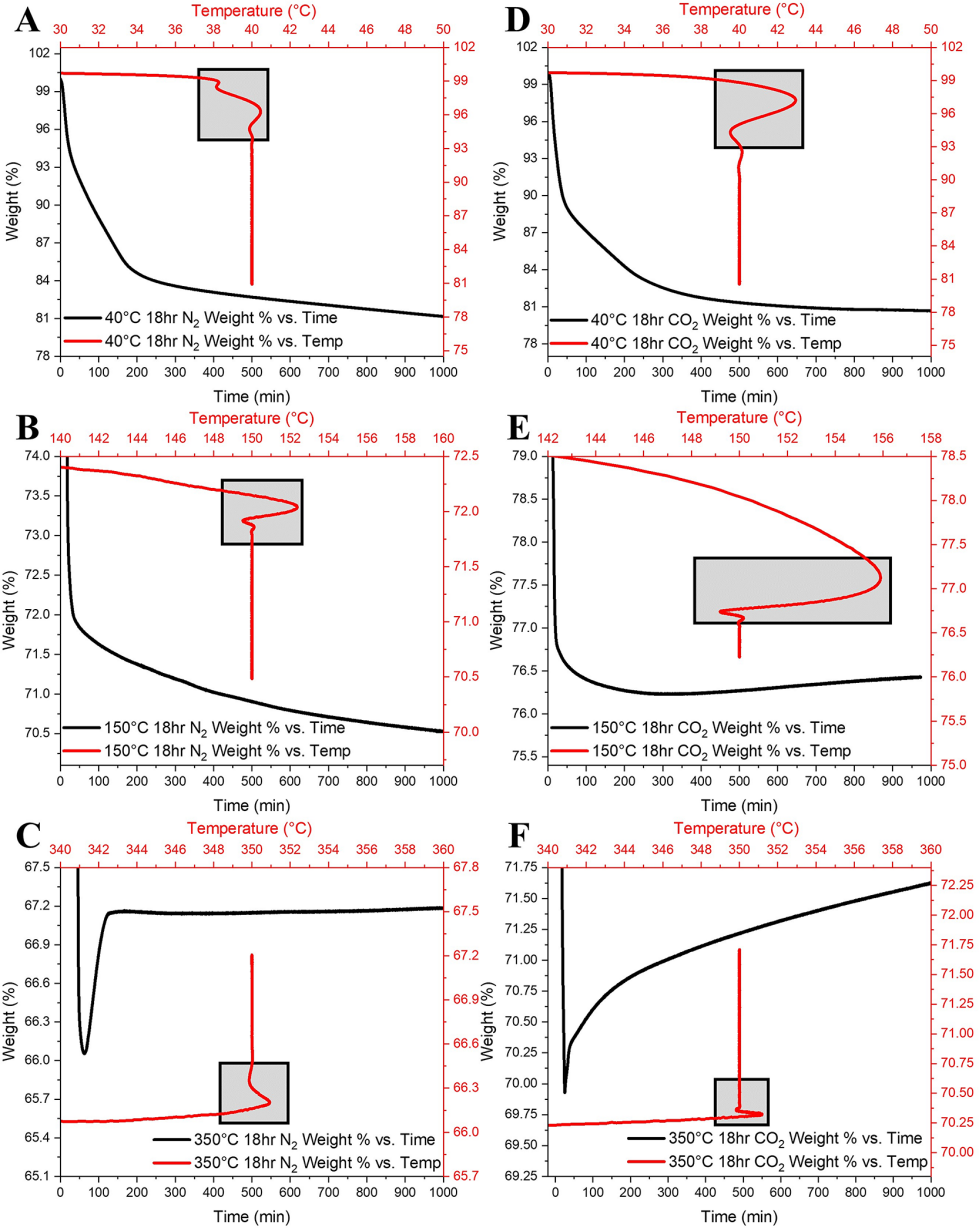
TGA curves (weight vs time in black; weight vs temperature in red) of the **KUPS-1** material under an N_2_ atmosphere held isothermally at (A) 40 °C, (B) 150 °C, and (C) 350 °C and under a CO_2_ atmosphere held isothermally at (D) 40 °C, (E) 150 °C, and (F) 350 °C. The gray box corresponds to the region where the temperature equilibration occurred within the TGA instrument.

The TGA curves of the **KUPS-1** material were recorded at a ramp rate of 5 °C/min until the target temperature (40, 150, or 350 °C) was reached within the instrument. The temperature was equilibrated, and the sample was held at the target temperature for 18 h with a continuous monitoring of sample weight (Figure 3). We hypothesized that the experiment carried out under a CO_2_ atmosphere would result in a significant carbon capture by the uranyl superoxide, resulting in elevated weight gain due to carbonate production, while the experiments carried out under an N_2_ atmosphere would show no significant weight gain. Initial isothermal experiments under an N_2_ atmosphere at 40 and 150 °C (Figure 3A,B) aligned well with our hypothesis, showing a subtle weight loss over 18 h following equilibration of the temperature (gray box). However, the N_2_ isothermal experiment at 350 °C (Figure 3C) showed a 1% weight gain by the material over the course of 1 h after temperature equilibration, attributed to the trace levels of CO_2_ in the gas line. This was then followed by no changes in weight for the remaining 17 h of the experiment. Interestingly, the thermal behavior of **KUPS-1** under a CO_2_ atmosphere mimicked the findings from the N_2_ atmosphere series. **KUPS-1** held isothermally at 40 °C under CO_2_ (Figure 3D) displayed behavior identical with that of the same experiment carried out under N_2_, with a subtle weight loss over the 18 h of the experiment. At 150 °C the material showed a gradual weight loss during the first 5 h of the isothermal experiment under CO_2_ and displayed a weight gain for the last 13 h of the experiment (Figure 3E), which differed from the results observed under the N_2_ gas. The most intriguing results came from the TGA curve of the **KUPS-1** material held at 350 °C, showing an immediate weight gain once the temperature was equilibrated. After 3 h from the start of the experiment, a constant weight gain was observed at a rate of 0.0012% (±9.85 × 10^–7^) per minute that lasted throughout the 15 h of the experiment, suggesting a continuous uptake of CO_2_ gas by the material at 350 °C. Such a steady weight gain is equivalent to absorption of ∼0.3 mol of CO_2_ per day in the carbon dioxide bearing environments. These findings suggest that the reactivity of the superoxide anion is increasing with temperature, resulting in significant levels of carbon capture by the **KUPS-1** material, especially at the temperature ranges relevant to the surface of spent nuclear fuel.

**Figure 4:**
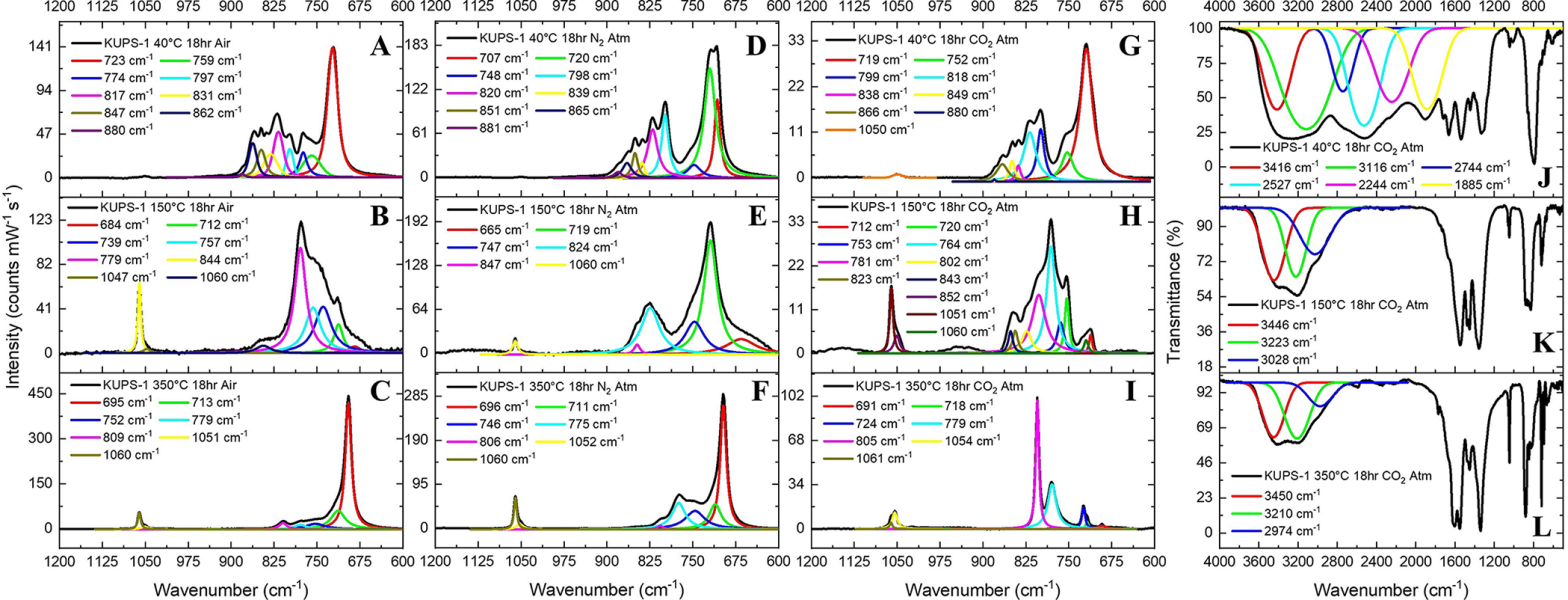
Fitted Raman spectra of the **KUPS-1** material held isothermally at (A) 40 °C, (B) 150 °C, and (C) 350 °C in open air, at (D) 40 °C, (E) 150 °C, and (F) 350 °C under an N_2_ atmosphere, and at (G) 40 °C, (H) 150 °C, and (I) 350 °C under a CO_2_ atmosphre. IR spectra of the **KUPS-1** material held isothermally at (J) 40 °C, (K) 150 °C, and (L) 350 °C under a CO_2_ atmosphere.

To definitively probe the chemistry of the superoxide anion at elevated temperatures, we performed Raman, IR (for select samples), and PXRD measurements on the resulting powders following the isothermal experiments in N_2_, CO_2_, and ambient air atmospheres (Figure 4). The Raman spectra of the **KUPS-1** material heated in the furnace exposed to open air (Figure 4A–C) showed that initially (40 °C) there are no significant changes in the uranyl speciation due to the ν_1_ uranyl symmetric stretch remaining at 723 cm^–1^. The prolonged heating of the sample at 40 °C showed an ingrowth of two new vibrational bands at 759 and 774 cm^–1^; however, the rest of the vibrational bands at 797–880 cm^–1^ matched well (±3 cm^–1^) the bands expected for pristine **KUPS-1**. The Raman spectrum of the powder following heating at 150 °C for 18 h (Figure 4B) showed significant chemical changes within the material due to the ingrowth of bands in the 694–757 cm^–1^ region associated with the degradation observed in the initial TGA experiment. A weak band at 844 cm^–1^ suggested the presence of the O_2_^2–^ ligand bound to the uranyl, while all other vibrational bands associated with the superoxide/peroxide ligands disappeared from the spectrum. Elevated temperature enhanced the reactivity of the superoxide ligand, causing it to react with carbon dioxide in open air, resulting in the presence of carbonate on the surface of the material, as demonstrated by the bands at 1047 cm^–1^ (very weak) and 1060 cm^–1^ (strong). The two bands corresponded to the ν_4_ mode of the CO_3_^2–^ bound to uranyl and potassium, respectively; however, the K_2_CO_3_ phase is present in significantly greater quantities on the basis of the normalized intensity data. Following a 350 °C isothermal experiment for 18 h (Figure 4C), major vibrational features were located at 695 and 779 cm^–1^ corresponding to the presence of potassium uranate, followed by bands at 713 and 809 cm^–1^ that indicate residual uranyl superoxide and uranyl tricarbonate phases. The presence of the carbonate was also confirmed by bands at 1051 and 1060 cm^–1^ as a result of the ν_4_ mode of the CO_3_^2–^ anion and at 752 cm^–1^ that can be assigned to the CO_3_^2–^ bound to the uranyl cation. The overall trend seen in the Raman spectra of the thermal series carried out in open air shows that at temperatures above 40 °C the peroxide ligands tend to break down, while the superoxide ligands, in turn, have an enhanced reactivity, resulting in a significant proportion of potassium carbonate with smaller amounts of uranyl carbonates. This trend was further confirmed in the Raman spectra of the N_2_ gas series, as in the Raman spectrum of the solid material after it was held isothermally at 40 °C (Figure 4D), the majority of vibrational bands match to those of the starting material **KUPS-1**, except for the bands at 707 and 748 cm^–1^, which both suggest a slight change in the coordination environment around the uranyl. The isothermal 150 °C N_2_ experiment (Figure 4E) paralleled the spectrum obtained in open air, showing a significant ingrowth of carbonate on the basis of a band at 1060 cm^–1^ (ν_4_ CO_3_^2–^), suggesting that the carbonate is bound to the potassium rather than a uranyl. Additional modes are located at 665, 747, and 824 cm^–1^, but their assignments are not clear. Finally, the Raman spectrum of the material obtained after isothermal heating at 350 °C under an N_2_ atmosphere (Figure 4F) contained features similar to those obtained under identical experimental conditions in open air, showcasing the prevalence of potassium uranate and carbonate phases mixed with minor amounts of uranyl carbonates. The ingrowth of carbonate within the materials obtained under an N_2_ atmosphere could also have been possible due to trace-level CO_2_ within the N_2_ line or during the brief sample preparation for further analysis (Raman and PXRD), which was done under ambient conditions.

Similar transformations of **KUPS-1** material were noted in the Raman spectra following isothermal experiments at 40 and 150 °C (Figure 4G,H) carried out under CO_2_ atmosphere; however, the Raman spectrum for the solid held isothermally at 350 °C under CO_2_ displayed different vibrational signatures. Figure 4I shows that the high-intensity vibrational bands at 805, 691, 718, 724, and 1054 cm^–1^ match well the bands predicted for the potassium uranyl tricarbonate species. There is additional potassium carbonate present, as evidenced by the band at 1061 cm^–1^, and a small peak at 691 cm^–1^ may correspond to minor amounts of potassium uranate. The Raman spectrum of **KUPS-1** held isothermally at 350 °C under a CO_2_ atmosphere was the first time the 1054 cm^–1^ band obtained higher normalized intensity in comparison to the band at 1061 cm^–1^, suggesting a prevalence of uranyl carbonate over the potassium carbonate phase under these conditions.

Infrared spectroscopy of the CO_2_ atmosphere experimental series (Figure 4J–L) further confirmed that the bulk of the **KUPS-1** material held isothermally at 40 °C does not undergo significant speciation changes on the basis of the overlapping bands in the 1880–4000 cm^–1^ region corresponding to ν_O–H_ vibrations of H_2_O, H_2_O_2_/OOH^–^, and O_2_^–^/OOH^•^, the majority of which disappear with an increase in temperature. The prominent band at 880 cm^–1^ in all IR spectra was assigned to the ν_3_ uranyl asymmetric stretch. Three vibrational bands at 695, 720, and 1047 cm^–1^ appear to grow in across the series from 40 to 350 °C corresponding to the CO_3_^2–^ deformation and stretching modes associated with the potassium uranyl tricarbonate phase. Thus, IR spectrscopy further confirms the elevated carbon capture by the **KUPS-1** material in the presence of CO_2_ at elevated temperatures. Unfortunately, the PXRD series showed that most of solids following the experiments are either amorphous or poorly crystalline mixed phases that cannot be well-matched to any literature pattern; however, the PXRD pattern of **KUPS-1** held isothermally at 350 °C for 18 h under a CO_2_ atmosphere matched a potassium uranyl tricarbonate phase (detailed PXRD studies can be found in the Supporting Information).

**Figure 5:**
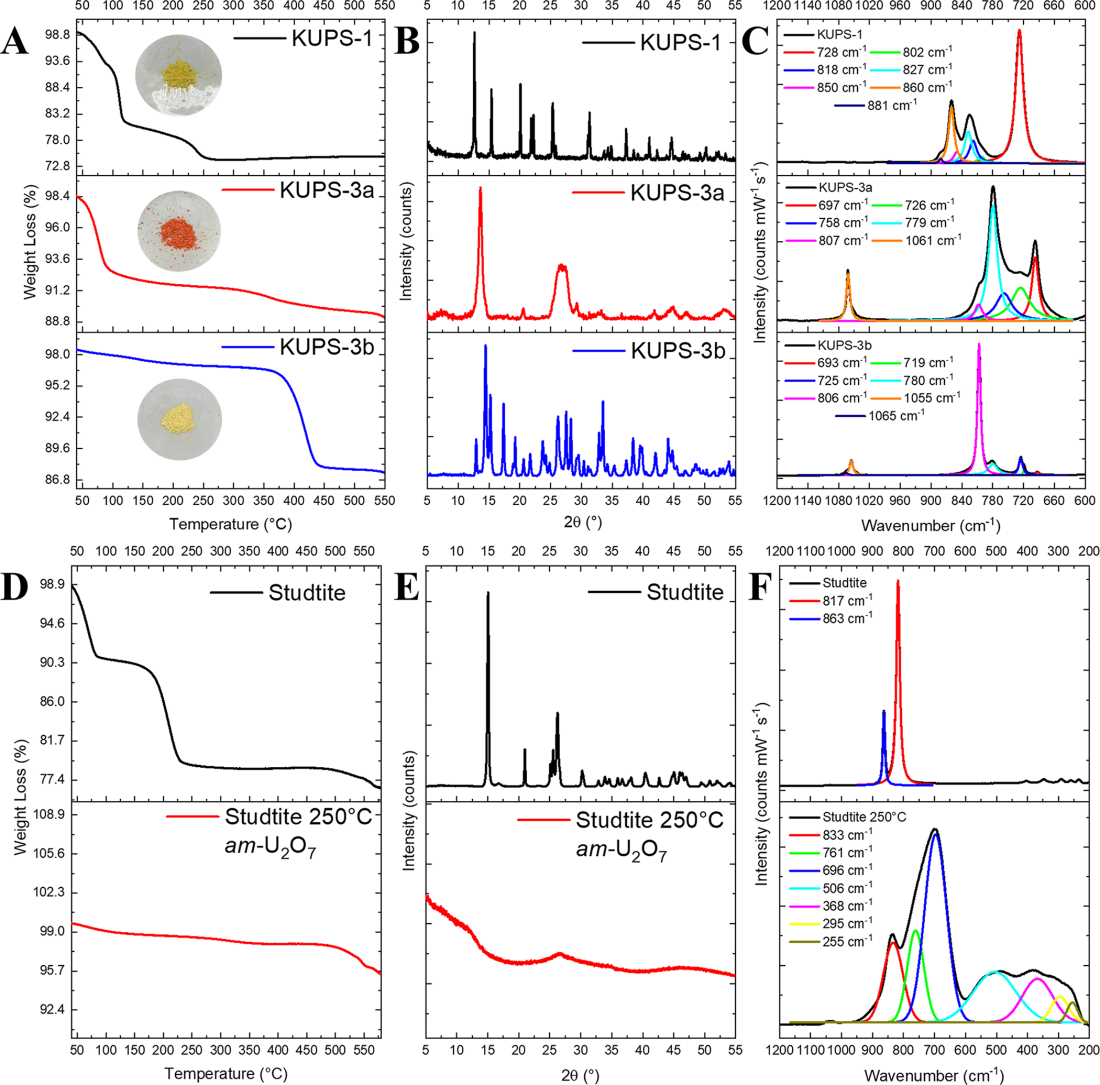
(A) TGA curve series and solid images of **KUPS-1**, **KUPS-3a** (**KUPS-1** heated at 250 °C for 4 h), and **KUPS-3b** (**KUPS-3a** exposed to air for 7 days). (B) PXRD pattern series of **KUPS-1**, **KUPS-3a**, and **KUPS-3b**. (C) Fitted Raman spectra series of **KUPS-1**, **KUPS-3a**, and **KUPS-3b**. (D) TGA curves for studtite and the amorphous U_2_O_7_ phase. (E) PXRD patterns of studtite and *am*-U_2_O_7_. (F) Fitted Raman spectra of studtite and *am*-U_2_O_7_.

Additionally, we carried out a comprehensive comparison of the thermal behavior between uranyl superoxide (**KUPS-1**) and studtite, a known alteration product of spent nuclear fuel. The decomposition pathway of studtite has been explored in detail and is due to the presence of the amorphous U_2_O_7_ intermediate appearing after the dehydration of metastudtite UO_2_(O_2_)·2H_2_O at 220 °C.^36,49,50^ The *am-*U_2_O_7_ intermediate was shown to be reactive in the presence of water, resulting in O_2_ gas evolution and formation of metaschoepite.^49^ The local structure of *am-*U_2_O_7_ has been extensively studied using vibrational spectroscopy, ^17^O NMR spectroscopy, powder X-ray diffraction, XANES, and DFT calculations;^51,52^ however, the exact structure of *am-*U_2_O_7_ is still unknown. The proposed mechanism of *am-*U_2_O_7_ formation involves cleavage of peroxide O–O bonds, aqua ligand U–O bonds, and certain uranyl U–O_ax_ bonds in metastudtite, which leads to an amorphous *am-*U_2_O_7_ phase, which in turn fuses into an α-UO_3_ crystalline phase at 535 °C.^50,53^ To compare and contrast the thermal behaviors of **KUPS-1** and studtite, we prepared pristine studtite according to the procedure described in the Experimental Methods and confirmed its purity using TGA, Raman spectroscopy, and PXRD (Figure 5D–F). We then followed a previously reported procedure to obtain *am-*U_2_O_7_ by heating studtite in the furnace at 250 °C for 4 h, followed by a full characterization of the resulting phase.^49^ The experimental steps and characterization were repeated with the **KUPS-1** material, resulting in an orange **KUPS-3a** phase immediately after heating at 250 °C for 4 h, which then transformed into the bright yellow solid **KUPS-3b** after exposure to air for 7 days (Figure 5A).

The orange **KUPS-3a** solid was an extremely hygroscopic powder, which accounts for the initial weight loss of surface-adsorbed water (∼6%) in the TGA curve of **KUPS-3a**. The PXRD pattern of **KUPS-3a** revealed two broad peaks centered at 14 and 26° 2θ, suggesting that the material was amorphous but contained ordered nanodomains. The Raman spectrum of **KUPS-3a** was similar to the vibrational spectrum of the **KUPS-1** solid heated isothermally for 18 h at 150 °C in open air (Figure 4B) with major vibrational features at 697, 726, 758, 779, 807, and 1061 cm^–1^. Raman spectra of the **KUPS-3a** phase suggest a mixed uranyl carbonate–potassium carbonate phase along with residual uranyl superoxide/peroxide, similar to those observed in isothermal experiments. Upon exposure of **KUPS-3a** to ambient atmosphere for 7 days, the yellow **KUPS-3b** solid was formed, with the experimental PXRD pattern matching that of K_4_[(UO_2_)(CO_3_)_3_]. The Raman spectrum of **KUPS-3b** also matched vibrational signatures of K_4_[(UO_2_)(CO_3_)_3_]with major bands at 806 cm^–1^ (ν_1_ U=O_ax_) and 1055 cm^–1^ (ν_4_ CO_3_^2–^ bound to U).

Results from the studtite dehydration experiment were similar to those previously reported in the literature^36^ but may also suggest a slight difference in the interpretation of the U_2_O_7_ phase. Investigating the PXRD pattern of *am-*U_2_O_7_, we observe a weak, broad feature centered around 26° 2θ seen in previous studies,^49^ which suggests a nanoscale domain of coherent diffraction for *am-*U_2_O_7_ but also matches with PXRD reports of am-UO_3_.^54^ The Raman spectrum of *am-*U_2_O_7_, however, shows vibrational modes spanning from 200 cm^–1^ up to 900 cm^–1^, which are distinct from the spectra of **KUPS-1**, **KUPS-3a**, and **KUPS-3b**. While bands at 833, 761, and 696 cm^–1^ could correspond to ν_1_ and ν_2_ modes of CO_3_^2–^, the lack of the strong ν_4_ mode and other deformations of CO_3_^2–^suggest that *am-*U_2_O_7_ is not reactive toward molecular CO_2_. The high-intensity band at 696 cm^–1^ likely corresponds to the ν_1_ uranyl symmetric stretch, and that at 833 cm^–1^ is likely associated with the ν_O–O_ stretch of the peroxide ligand. Additional bands located between 255 and 506 cm^–1^ are typically observed for U^VI^ oxide phases and may be indicative of amorphous UO_3_ according to literature reports.^55,56^ Overall, the *am-*U_2_O_7_ phase exhibits distinct vibrational signatures and chemical behavior favoring the formation of metaschoepite (UO_2_)_8_O_2_(OH)_12_·10H_2_O, versus a metastable uranyl superoxide **KUPS-1** phase that favors a formation of uranyl tricarbonates, such as agricolaite K_4_(UO_2_)(CO_3_)_3_.

We note that the reactivity of the amorphous U_2_O_7_ phase to form metaschoepite deserves additional attention to further delineate the nature and reactivity of the solid. One item to be considered is that the reaction of U_2_O_7_ with water to form metaschoepite and O_2_ gas does not result in a balanced chemical and reduction–oxidation reaction. Additional hydrogen needs to be added to the product to balance both the redox and overall chemical reactions. The second thing that could be considered is the possibility that the *am-*U_2_O_7_ phase is a mixture of amorphous UO_3_ and hypothetical dehydrated studtite UO_2_(O_2_), resulting in a combined stoichiometry of U_2_O_7_. This is also supported by previous literature by Cordfunke,^57,58^ Sato,^38^ and colleagues which indicated that the decomposition of metastudtite is an endothermic reaction that results in the formation of amorphous UO_3_. Pure amorphous UO_3_ is typically prepared by calcining studtite at 400 °C for 8 h;^54^ thus, the heating observed within the TGA experiment may result in incomplete decomposition and formation of a binary phase. In addition, Kirkegaard et al. noted that metaschoepite is formed from reacting amorphous UO_3_ (dark orange color) with water at 80 °C to form the bright yellow metaschoepite.^59^ If U_2_O_7_ is a binary mixture, the peroxide phase could be responsible for the evolved gas, whereas the color change and ultimate identity of the product in water could be related to the presence of amorphous UO_3_. Additional investigations could provide a more mechanistic understanding of the process that would improve our fundamental knowledge of U^VI^ phases containing reactive oxygen species.

## 4 Conclusion

Extensive TGA, DSC, Raman, and PXRD studied were performed on the uranyl superoxide material **KUPS-1** to probe the thermodynamics, stability, and chemical behavior of uranyl superoxide at elevated temperatures under open air, N_2_, and CO_2_ environments. The DSC results demonstrated that the **KUPS-1** material is metastable, showing two exothermic reactions with −148.78 ± 4.27 and −29.04 ± 1.55 kJ/mol at 90 and 240 °C, respectively. With an increase in temperature, the superoxide ligand coordinated to the uranyl within the **KUPS-1** material does not break down but, instead, is enhanced in reactivity with carbon dioxide at a temperature >350 °C across all experimental conditions (open air, N_2_ atmosphere, and CO_2_ atmosphere). The selectivity and sensitivity of uranyl superoxide toward CO_2_ is extraordinary, especially in N_2_ environments where carbon dioxide is only present in trace-level quantities.

The tranformation of the uranyl superoxide **KUPS-1** phase into a uranyl tricarbonate phase (in our case, K_4_[UO_2_(CO_3_)_3_] or the mineral agricolaite) at the temperature ranges associated with spent nuclear fuel provides an important insight into our understanding of how surface alteration phases are being formed. Uranyl carbonates, such as rutherfordine UO_2_CO_3_ and cejkaite Na_4_[UO_2_(CO_3_)_3_], are among the most common and identifiable alteration phases of the nuclear waste, the latter being isostructural with agricolaite K_4_[UO_2_(CO_3_)_3_]. The overall reactivity of the uranyl superoxide material **KUPS-1** in the presence of molecular CO_2_ leads to the formation of a primary potassium carbonate phase at 150 °C, which transforms into an agricolaite mineral phase at a temperature of 350 °C or upon exposure of the material to ambient atmosphere for 1 week, suggesting that tetrapotassium uranyl tricarbonate is a thermodynamically stable product.

Our findings present a possible link between a continuous α-radiolysis on the surface of nuclear waste, leading to the formation of uranyl superoxide, with the subsequent reactivity of the superoxide at elevated temperatures (>350 °C) of the fuel rods, resulting in extensive uranyl carbonate phases. While the current study shows the reactivity of the uranyl superoxide phase at elevated temperatures, we acknowledge that we do know know if the superoxide phase will form at these high temperatures. In addition, there are other radical pathways that could lead to the formation of other secondary alteration phases, including other uranyl peroxide and oxyhdroxide phases. Additional studies are necessary to evaluate the effect of these reactive oxygen species on the nuclear materials and provide a mechanistic understanding of the effects of free radicals on actinide chemistry.

## Supporting Information Available

Crystallographic information and vibrational spectroscopy of the uranyl superoxide material **KUPS-1**, processed and raw powder X-ray diffraction patterns of **KUPS-1** following heating at various temperatures (40, 150, 350 °C) and under various atmospheres (open air, N_2_, CO_2_), fitted Raman spectra of solid-state samples of **KUPS-1** following heating at various temperatures (40, 150, 350 °C) and under various atmosphere (open air, N_2_, CO_2_), experimental thermogravimetric analysis curves, differential scanning calorimetry mesurements, and enthalpy calculations for the **KUPS-1** material (PDF)
