# Supplementary material for: Thermodynamics and Chemical Behavior of Uranyl Superoxide at Elevated Temperatures
Source: ACS Mater Au. 2021 Aug 26;2(1):33–44. doi: 10.1021/acsmaterialsau.1c00033 (PMC9928197; doi:10.1021/acsmaterialsau.1c00033)
Supplement: Supplementary file 1 — mg1c00033_si_001.pdf [file mg1c00033_si_001.pdf]

Supporting Information

Thermodynamics and Chemical Behavior of Uranyl Superoxide at Elevated Temperatures

Dmytro V. Kravchuk and Dr. Tori Z. Forbes*

Department of Chemistry, University of Iowa, Iowa City, Iowa 52242, United States

* Correspondence to: tori-forbes@uiowa.edu

Table of Contents

Background Information on KUPS-1

Figure S1. Ball and stick model of the uranyl peroxo/superoxo monomer 3
Table S1. Crystallographic information and structure refinement details for **KUPS-1** 4
Figure S2. Fitted Raman spectrum of pristine **KUPS-1** 5
Figure S3. Raman fitting statistics and parameters for **KUPS-1** 6
Figure S4. Infrared spectrum of **KUPS-1** 7
Figure S5. Experimental and calculated PXRD patterns of **KUPS-1** 8

Powder X-ray Diffraction

Figure S6: PXRD patterns of **KUPS-1** from **Figure 2** in the main text 9
Figure S7: Raw PXRD patterns of **KUPS-1** from **Figure 2** in the main text 10
Figure S8: PXRD patterns of **KUPS-1** 40°C 18hr under air, N_2, CO_2 11
Figure S9: PXRD patterns of **KUPS-1** 150°C 18hr under air, N_2, CO_2 12
Figure S10: PXRD patterns of **KUPS-1** 350°C 18hr under air, N_2, CO_2 13
Figure S11: PXRD comparison of **KUPS-1** 350°C 18hr CO_2, **KUPS-3b**, and agricolaite 14
Figure S12: Raw PXRD patterns of **KUPS-1** 40°C, 150°C, and 350°C 18hr open air..............15
Figure S13: Raw PXRD patterns of **KUPS-1** 40°C, 150°C, and 350°C 18hr CO_2 16
Figure S14: Raw PXRD patterns of **KUPS-1** 40°C, 150°C, and 350°C 18hr N_2 17

Solid-State Raman Spectroscopy

Figure S11: Raman fitting parameters of **KUPS-1** 40°C (**Figure 2**) 18
Figure S12: Raman fitting parameters of **KUPS-1** 150°C (**Figure 2**) 19
Figure S13: Raman fitting parameters of **KUPS-1** 350°C (**Figure 2**) 20
Figure S14: Raman fitting parameters of **KUPS-1** 40°C 18hr Air (**Figure 4A**) 21
Figure S15: Raman fitting parameters of **KUPS-1** 150°C 18hr Air (**Figure 4B**).......................22
Figure S16: Raman fitting parameters of **KUPS-1** 350°C 18hr Air (**Figure 4C**) 23
Figure S17: Raman fitting parameters of **KUPS-1** 40°C 18hr N_2 (**Figure 4D**).........................24
Figure S18: Raman fitting parameters of **KUPS-1** 150°C 18hr N_2 (**Figure 4E**).......................25
Figure S19: Raman fitting parameters of **KUPS-1** 350°C 18hr N_2 (**Figure 4F**).......................26

Figure S20: Raman fitting parameters of **KUPS-1** 40°C 18hr CO_2 (**Figure 4G**)........................27
Figure S21: Raman fitting parameters of **KUPS-1** 150°C 18hr CO_2 (**Figure 4H**)......................28
Figure S22: Raman fitting parameters of **KUPS-1** 350°C 18hr CO_2 (**Figure 4I**) 29

Thermodynamics Calculations

Figure S23: Thermogravimetric analysis of **KUPS-1** 30
Figure S24: DSC analysis of **KUPS-1** at 90 °C 31
Figure S25: DSC analysis of **KUPS-1** at 240 °C 32
Table 1: Average enthalpy data for **KUPS-1** at 90 °C 33
Table 2: Average enthalpy data for **KUPS-1** at 240 °C 33

Crystallographic Information

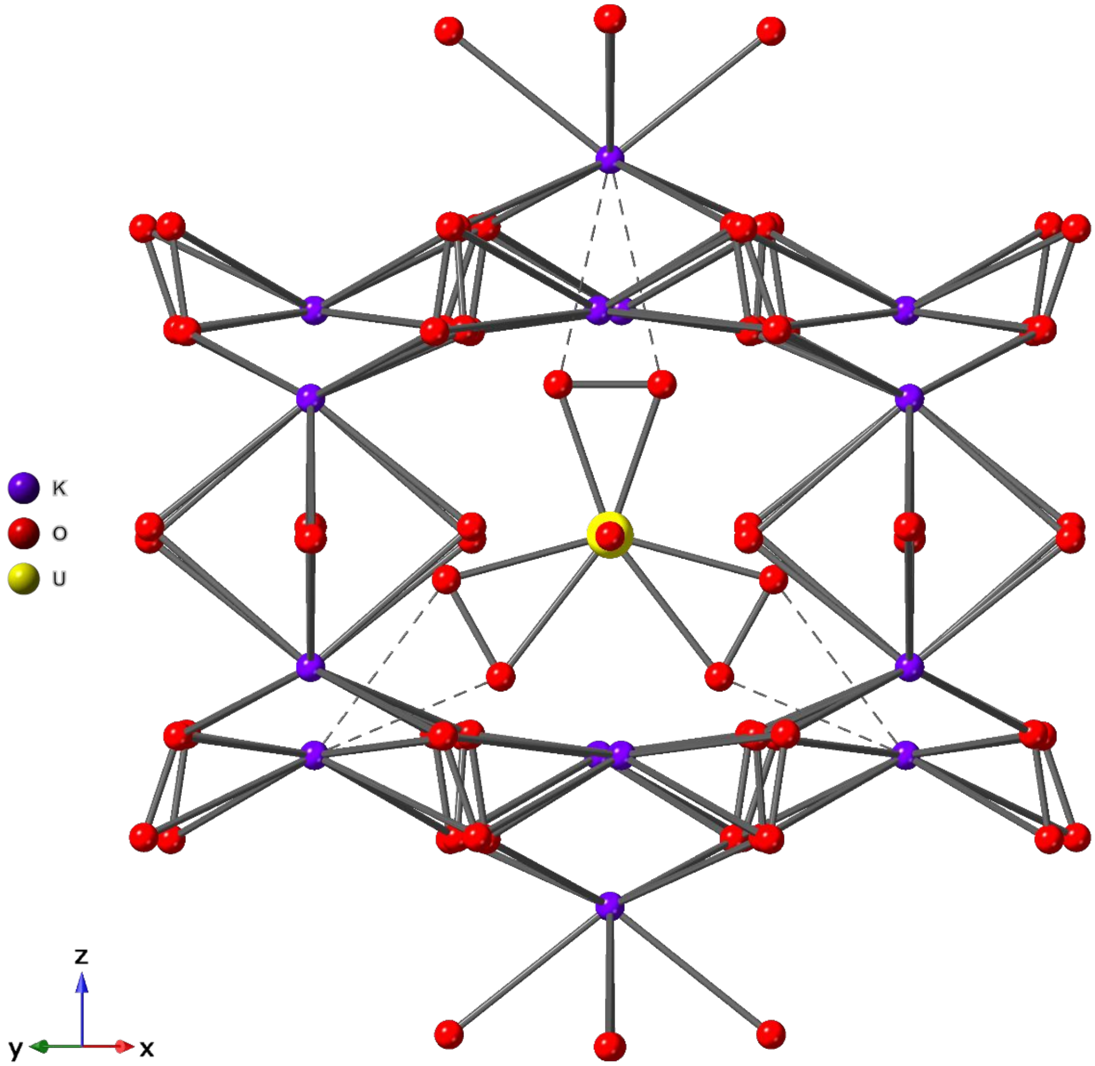

Figure S1: Ball and stick model of the uranyl peroxo/superoxo monomer inside the hydrate potassium hydrogen peroxide cage **KUPS-1**. Uranium, oxygen, and potassium atoms are depicted using yellow, red, and purple spheres respectively. Hydrogen atoms were omitted for clarity.

Table S2. Crystallographic information and structure refinement details for **KUPS-1** at 273K.

Identification code	KUPS-1
Empirical formula	$H_{16}K_4O_{20}U$
Formula weight	730.56
Temperature/K	273.15
Crystal system	Tetragonal
Space group	*I*4$_1$/*acd*
a/Å	11.4962(4)
b/Å	11.4962(4)
c/Å	27.5603(12)
α/°	90
β/°	90
γ/°	90
Volume/Å^3	3642.4(3)
Z	8
ρ_{calc}g/cm^3	2.664
μ/mm^{-1}	9.919
F(000)	2752.0
Crystal size/mm^3	0.75 × 0.5 × 0.5
Radiation	MoKα (λ = 0.71073)
2Θ range for data collection/°	5.818 to 50.7
Index ranges	-13 ≤ h ≤ 13, -13 ≤ k ≤ 13, -33 ≤ l ≤ 33
Reflections collected	39806
Independent reflections	838 [R_{int} = 0.0315, R_{sigma} = 0.0081]
Data/restraints/parameters	838/0/76
Goodness-of-fit on F^2	1.177
Final R indexes [I>=2σ (I)]	R_1 = 0.0286, wR_2 = 0.0613
Final R indexes [all data]	R_1 = 0.0356, wR_2 = 0.0651
Largest diff. peak/hole / e Å^{-3}	0.63/-1.05

The .CIF file with complete crystallographic information can be found in the Cambridge Crystallographic Data Center under deposition number 2047863.

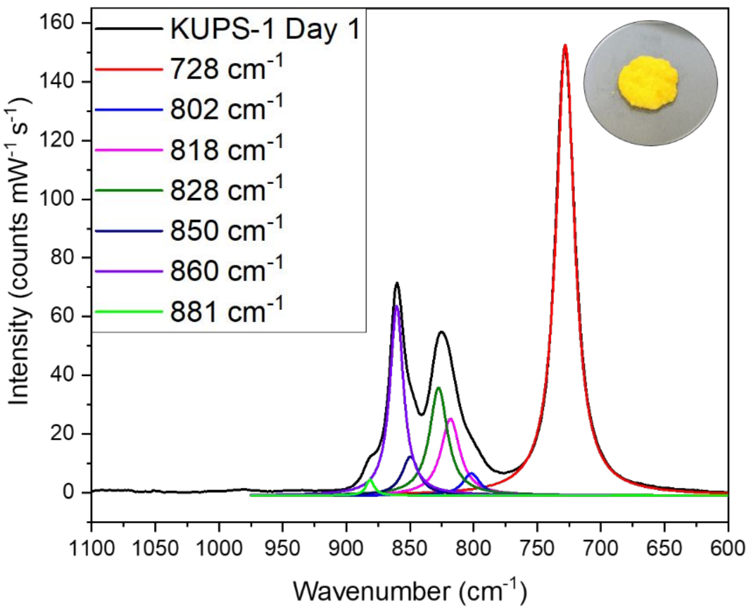

Raman Shift (cm^{-1})	Assignment
728	$\nu_1(UO_2^{2+})$
802	$\nu_{2/3}(O_2^{2-})$ bound to U
818	$\nu_2(O_2^{2-})$ bound to U
827	$\nu_3(O_2^{2-})$ bound to U
850	$\nu_1(O_2^{2-})$ bound to U
860	$\nu_1(O_2^{2-})$ bound to K
881	$\nu_1(O_2^{1-})$ bound to U

Figure S2: Fitted Raman spectrum of pristine **KUPS-1** in the spectral window of interest (600-1100 cm^{-1}). Color-coded vibrational bands noted with a peak centroid in the legend. Vibrational band assignments are summarized in the table on the right.

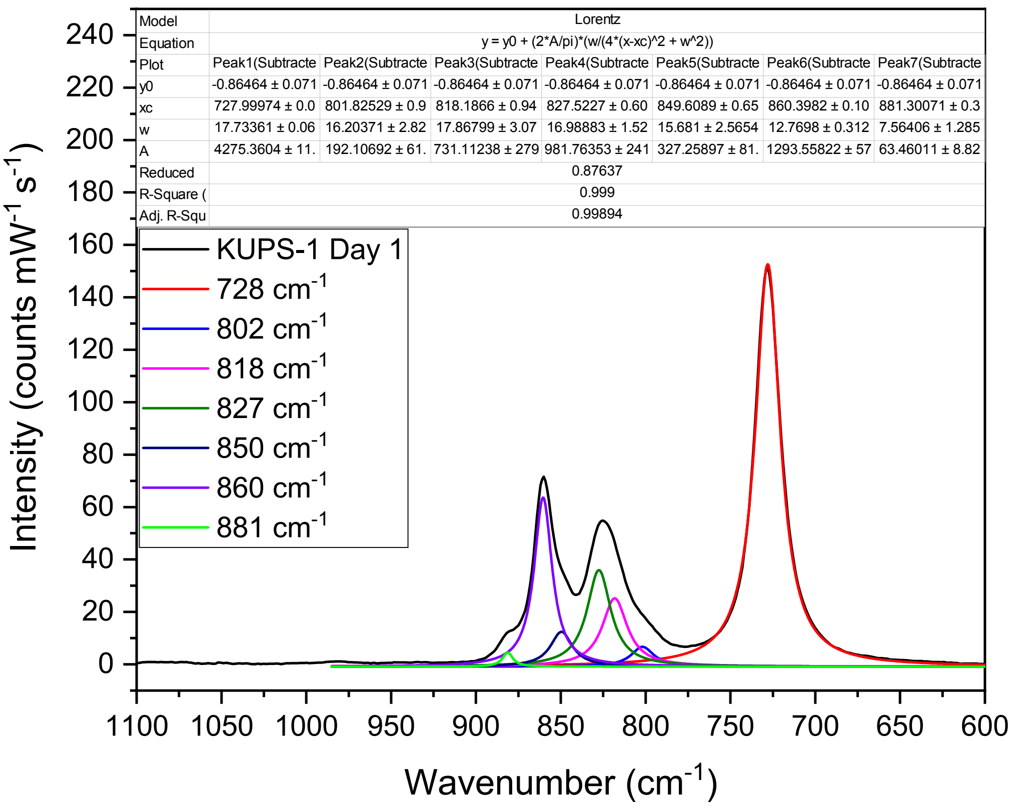

Figure S3: Fitted Raman spectrum of **KUPS-1** including fitting parameters, fitting function, peak centroids, FWHM information, and goodness of fit.

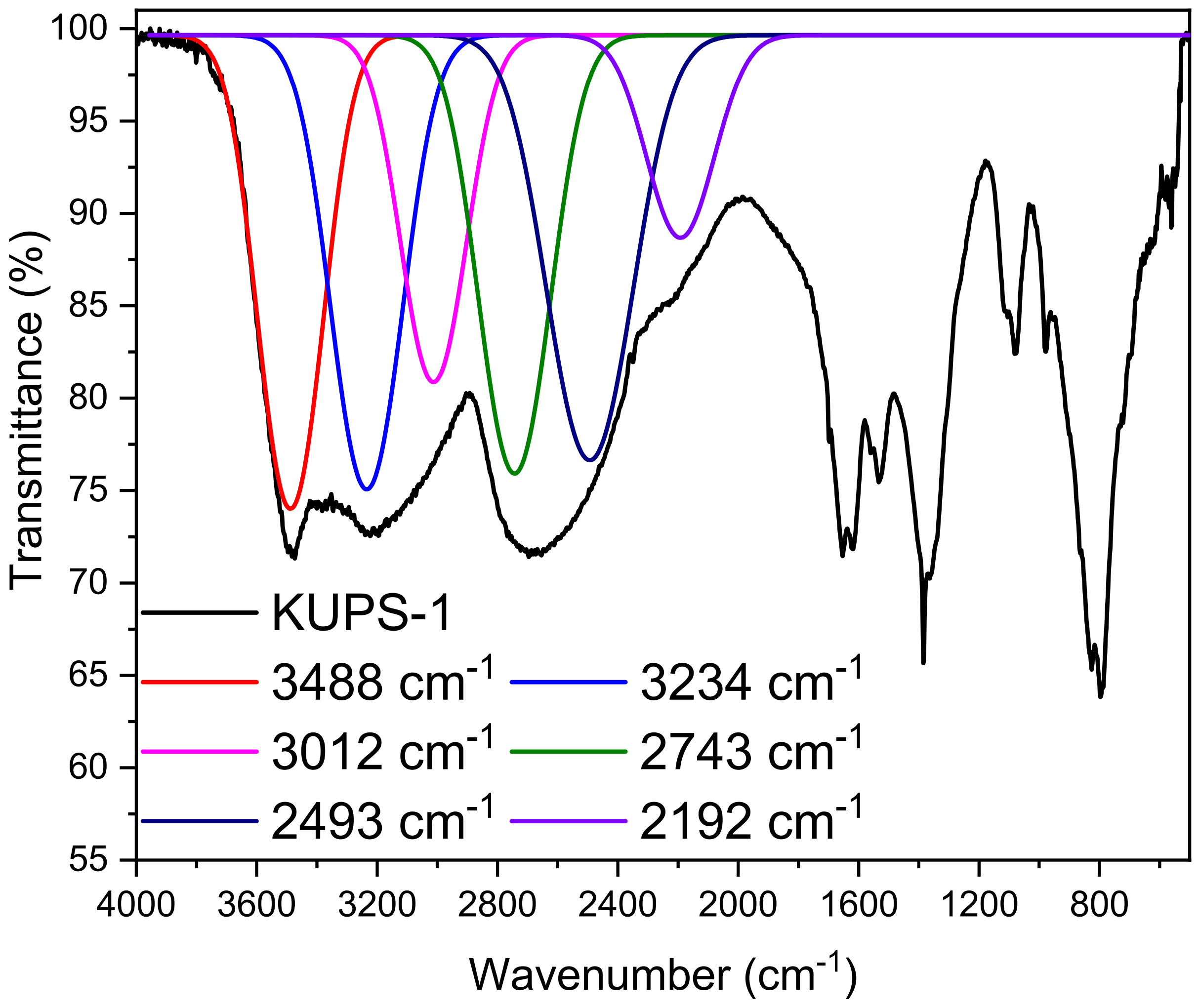

Figure S4: Infrared spectrum of **KUPS-1** compound with the fitted region between 2000 cm^{-1} and 4000 cm^{-1}.

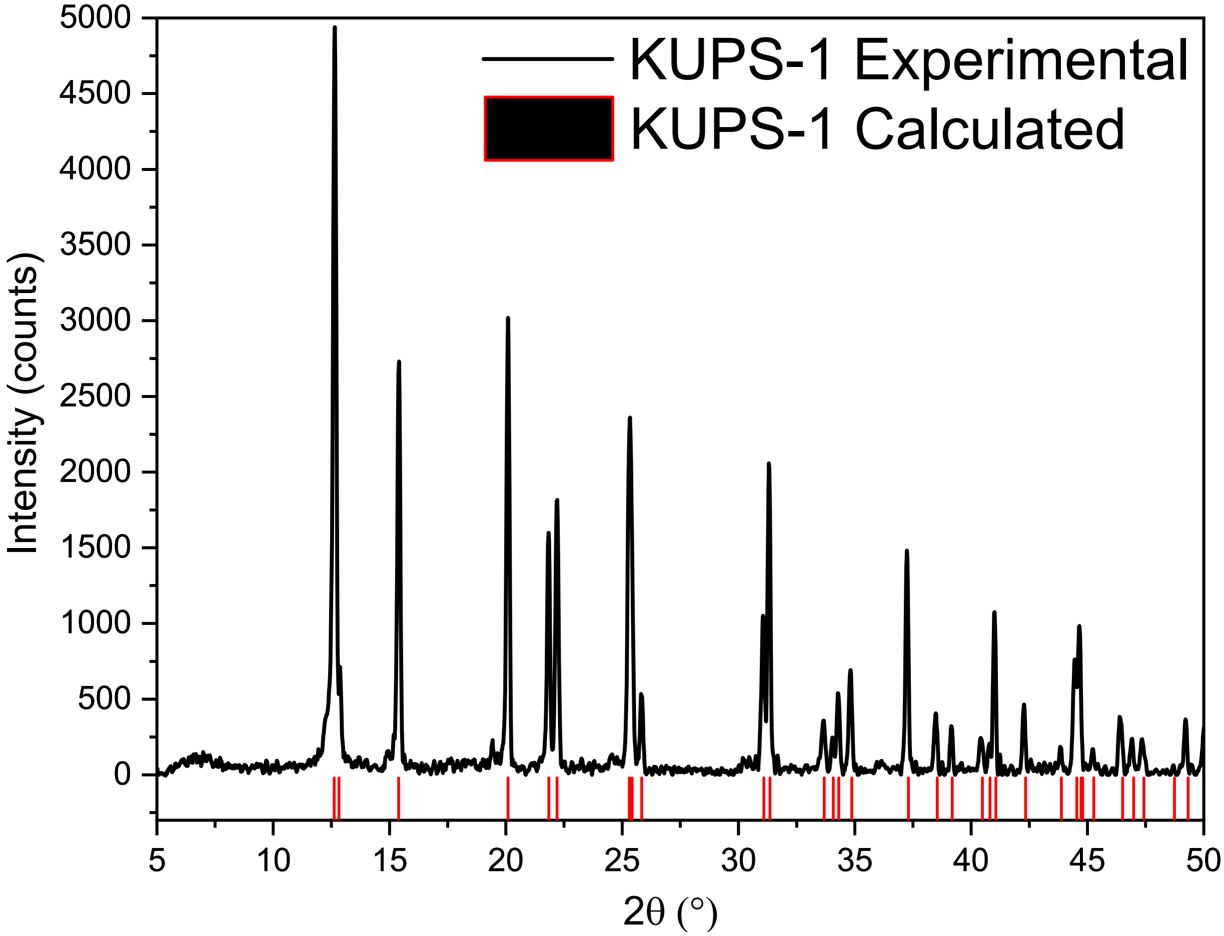

Figure S5: Experimental PXRD pattern of **KUPS-1** compound in black, aligned with the peaks from the calculated powder pattern using single-crystal data in red.

Powder X-ray Diffraction

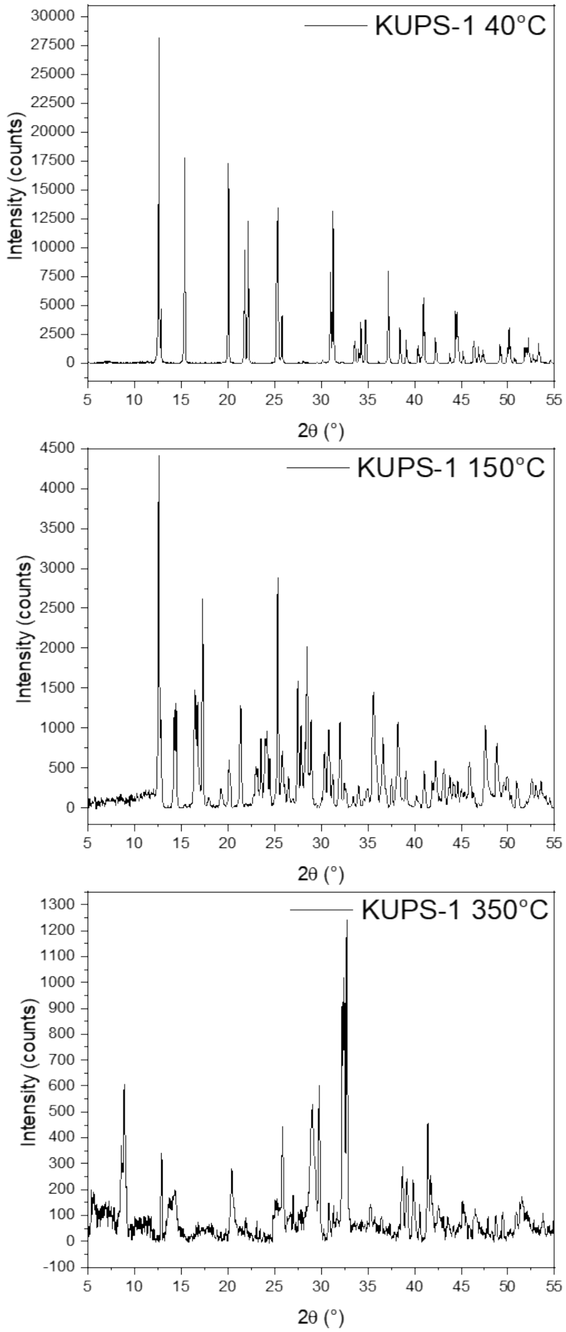

Figure S6: Experimental PXRD patterns of **KUPS-1** heated to 40 °C, 150 °C, and 350 °C enhanced from **Figure 2** in the main body of the text.

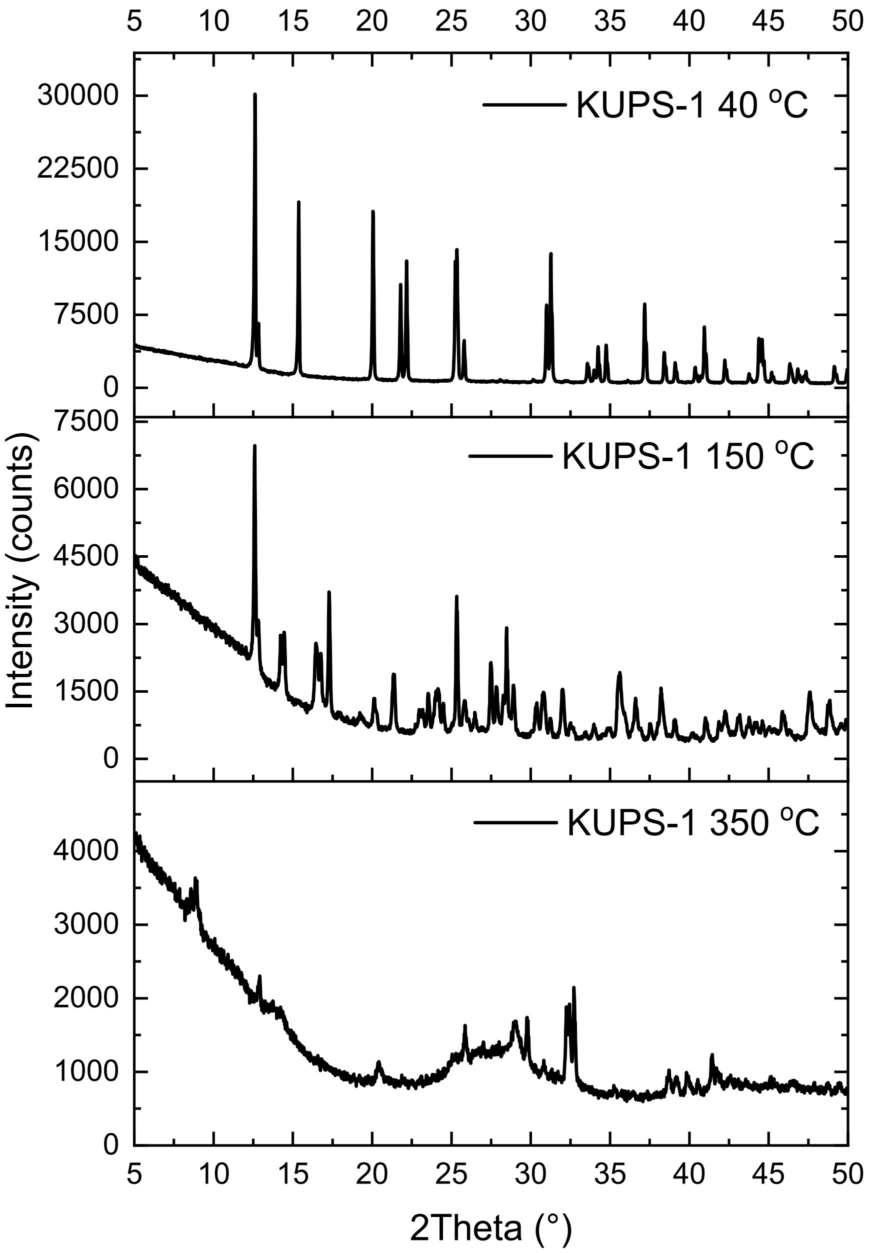

Figure S7: Unsubtracted/raw experimental PXRD patterns of **KUPS-1** heated to 40 °C, 150 °C, and 350 °C.

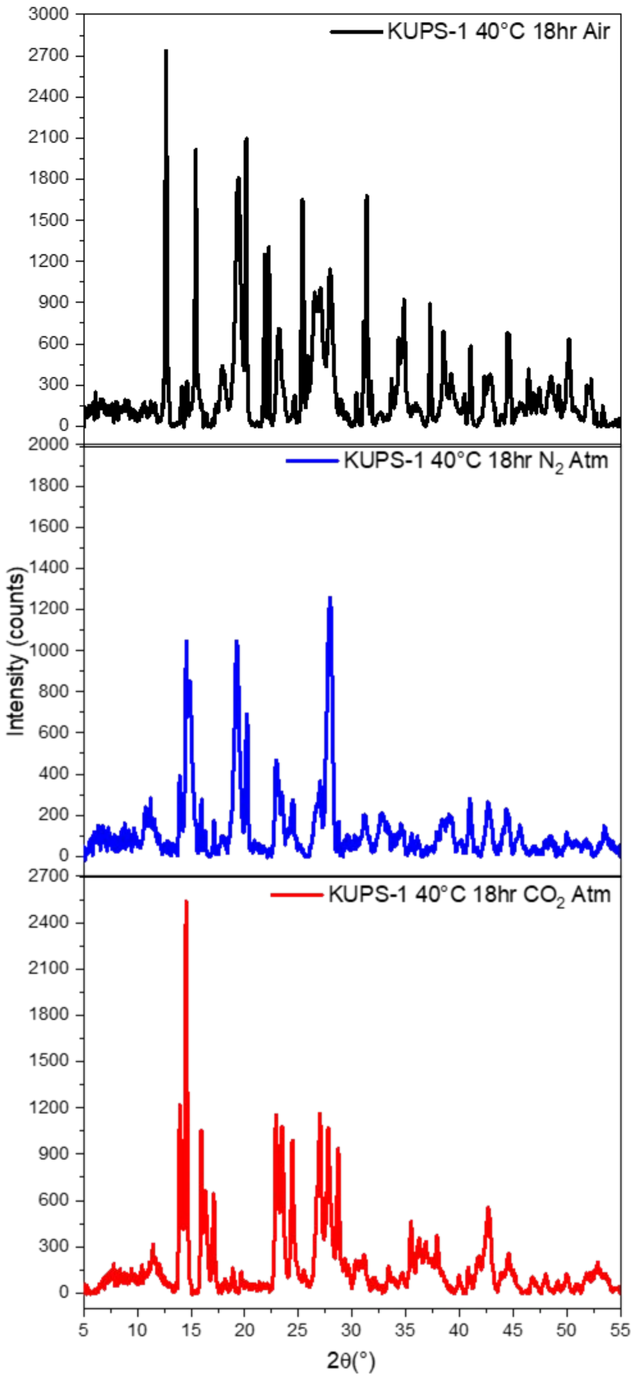

Figure S8: Experimental PXRD patterns of **KUPS-1** heated at 40 °C and held isothermal for 18 hours under open air, N_2 atmosphere, and CO_2 atmosphere.

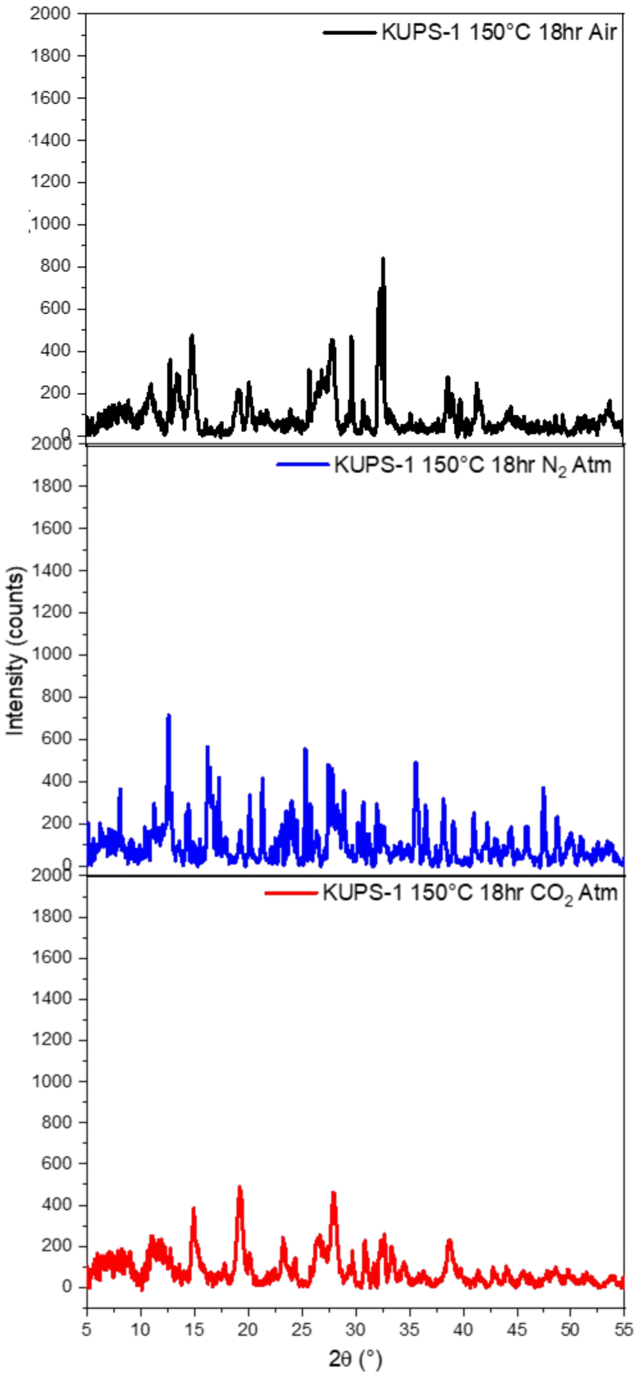

Figure S9: Experimental PXRD patterns of **KUPS-1** heated at 150 °C and held isothermal for 18 hours under open air, N_2 atmosphere, and CO_2 atmosphere.

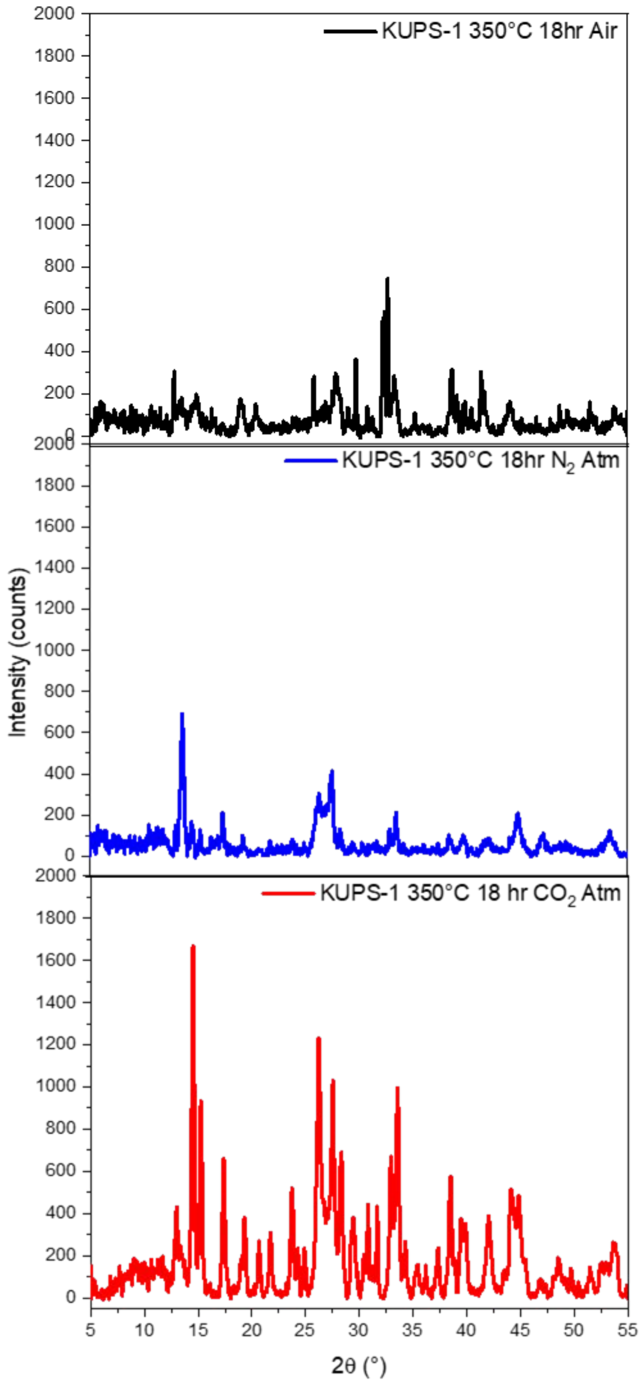

Figure S10: Experimental PXRD patterns of **KUPS-1** heated at 350 °C and held isothermal for 18 hours under open air, N_2 atmosphere, and CO_2 atmosphere**.**

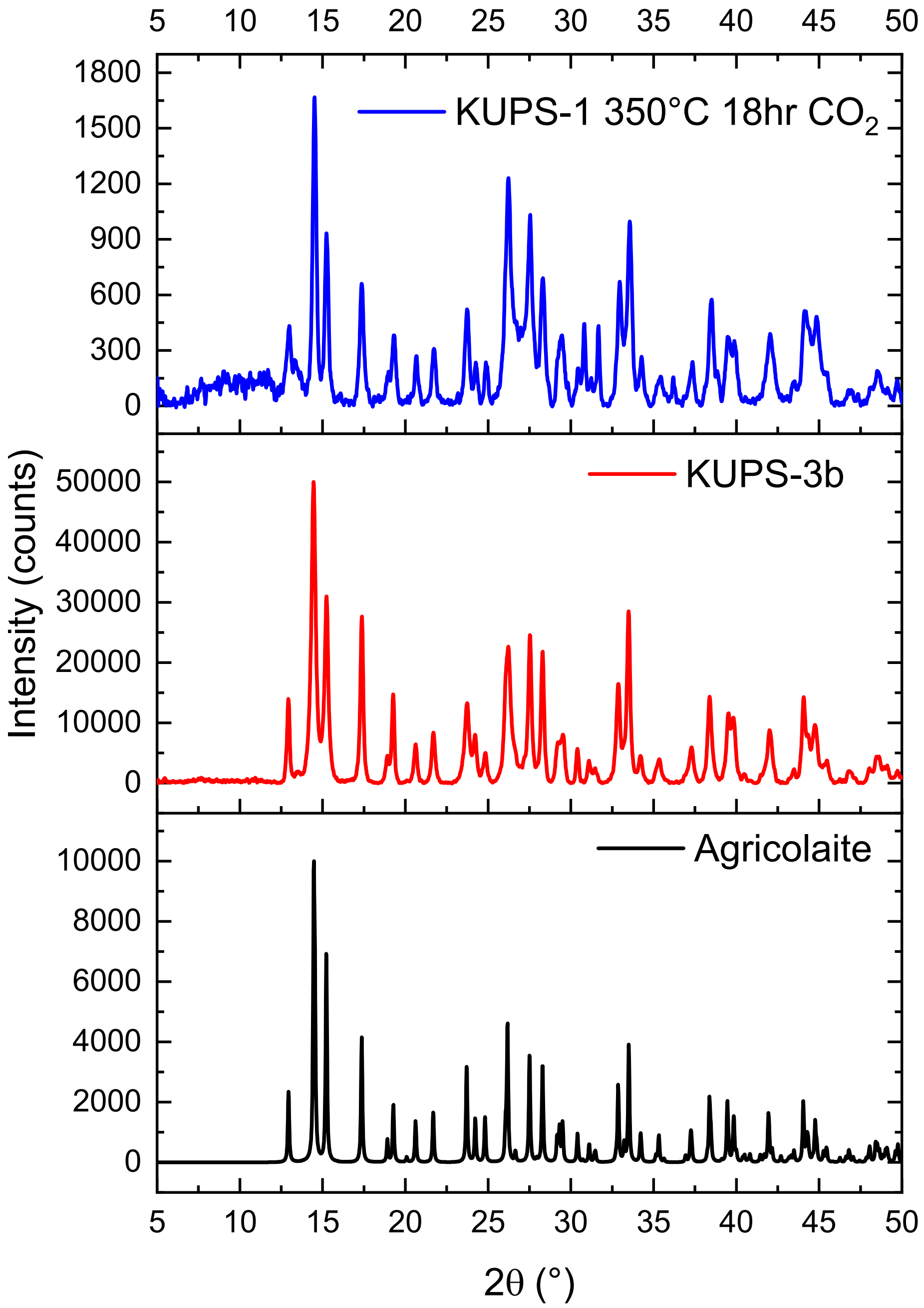

Figure S11: Experimental PXRD patterns of **KUPS-1** heated at 350 °C and held isothermal for 18 hours under CO_2 atmosphere blue, **KUPS-3b** in red, compared to literature patterns of agricolaite $K_4(UO_2)(CO_3)_3$ in black.

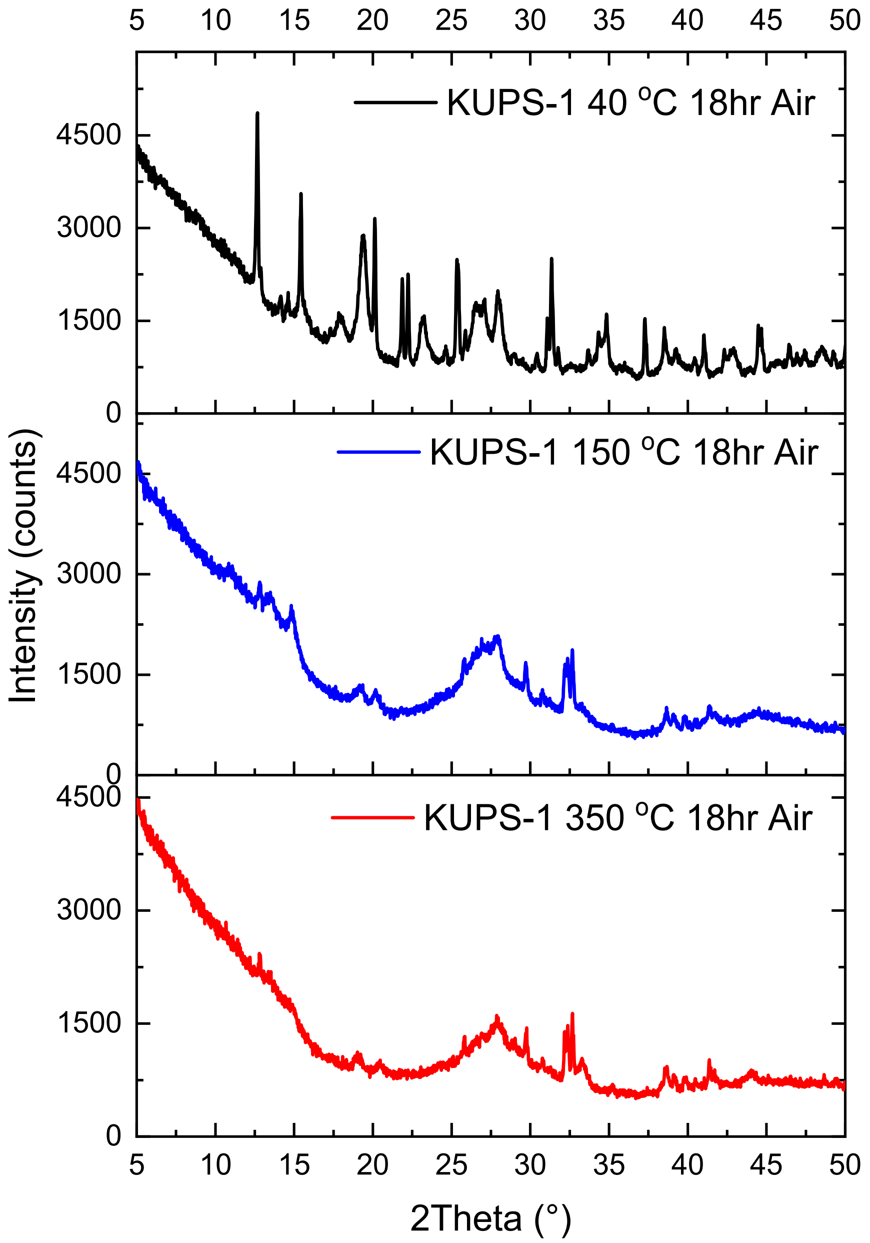

Figure S12: Unsubtracted/raw experimental PXRD patterns of **KUPS-1** heated at 40 °C, 150 °C, and 350 °C and held isothermal for 18 hours under open air atmosphere.

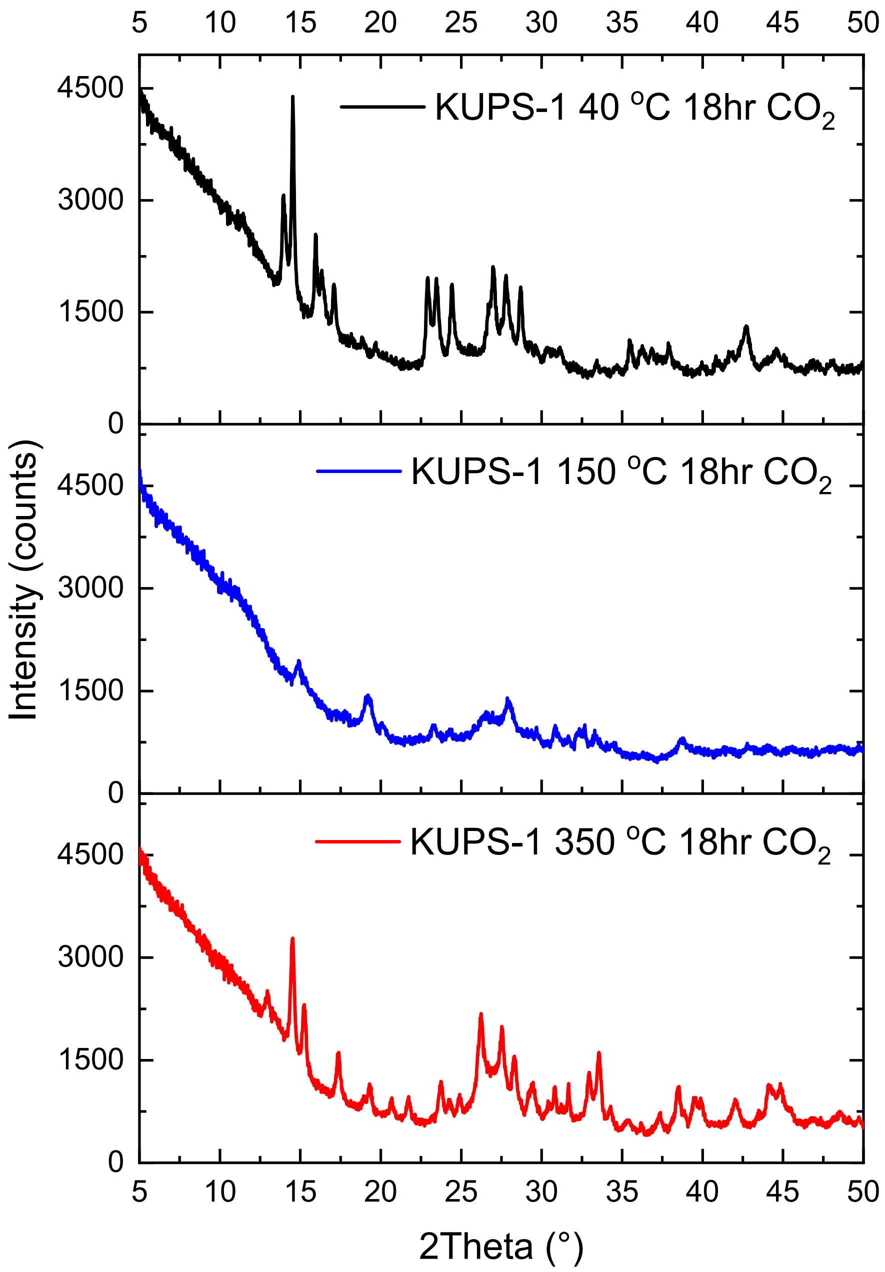

Figure S13: Unsubtracted/raw experimental PXRD patterns of **KUPS-1** heated at 40 °C, 150 °C, and 350 °C and held isothermal for 18 hours under CO_2 atmosphere.

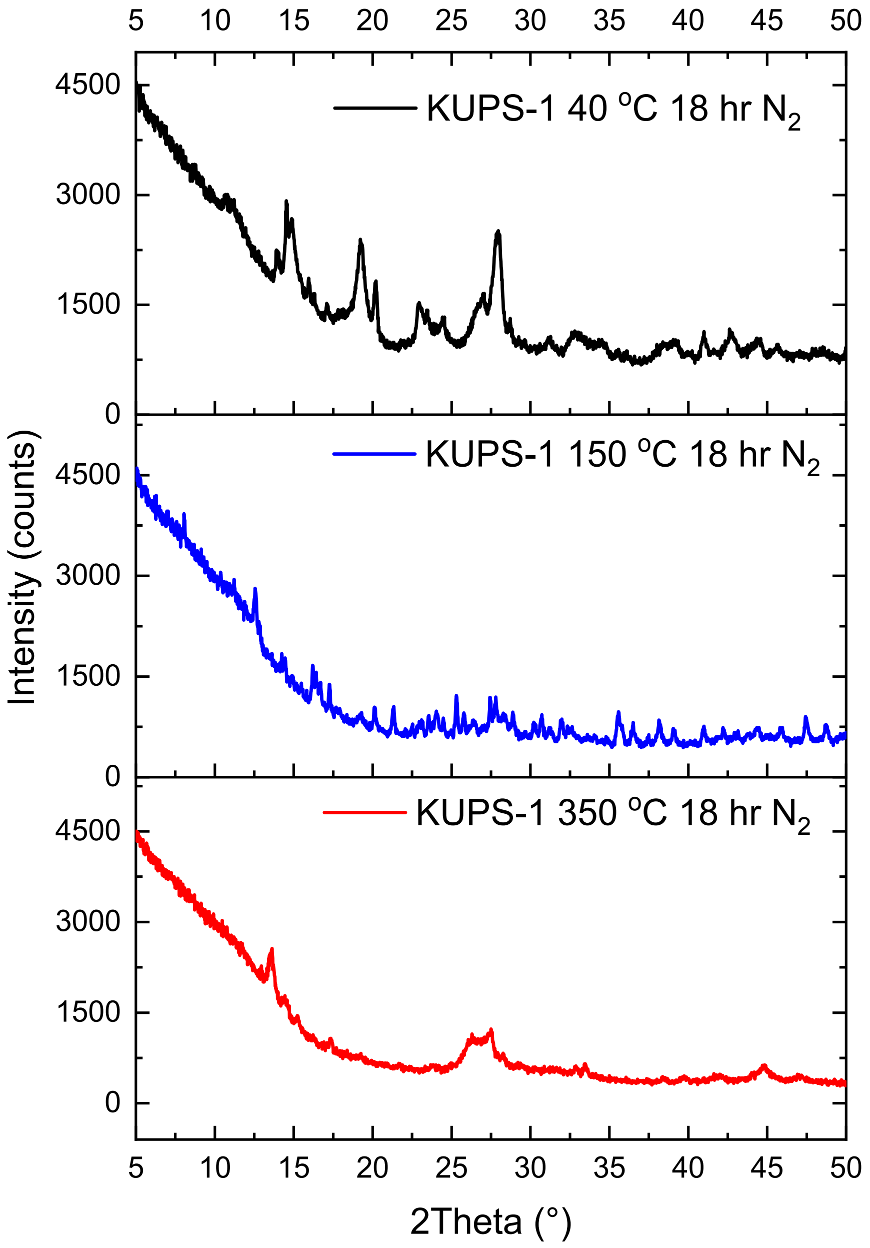

Figure S14: Unsubtracted/raw experimental PXRD patterns of **KUPS-1** heated at 40 °C, 150 °C, and 350 °C and held isothermal for 18 hours under N_2 atmosphere.

Solid-State Raman Spectroscopy

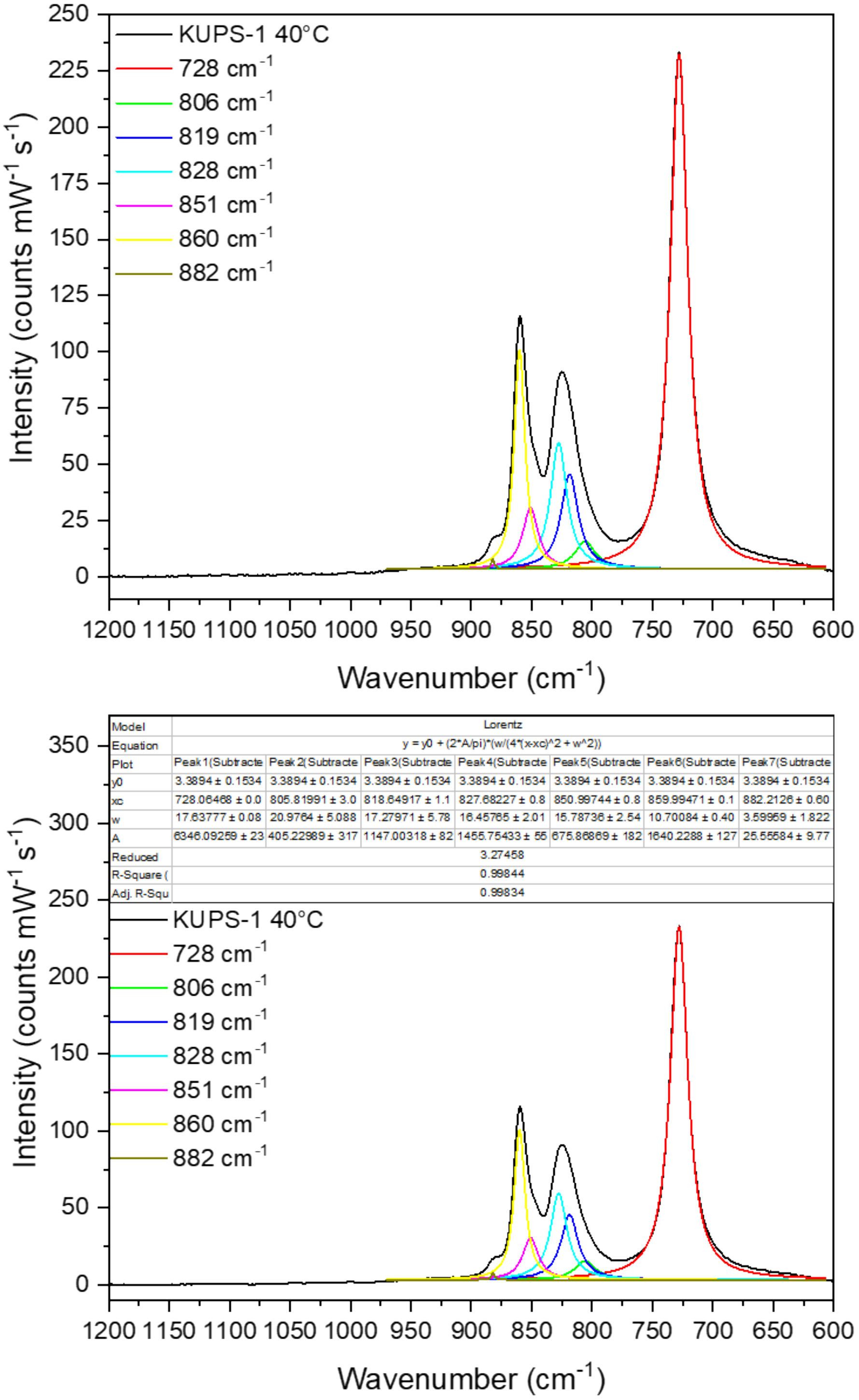

Model	Lorentz						
Equation	y = y0 + (2*A/pi)*(w/(4*(x-xc)^2 + w^2))						
Plot	Peak1(Subtracte	Peak2(Subtracte	Peak3(Subtracte	Peak4(Subtracte	Peak5(Subtracte	Peak6(Subtracte	Peak7(Subtracte
y0	3.3894 ± 0.1534	3.3894 ± 0.1534	3.3894 ± 0.1534	3.3894 ± 0.1534	3.3894 ± 0.1534	3.3894 ± 0.1534	3.3894 ± 0.1534
xc	728.06468 ± 0.0	805.81991 ± 3.0	818.64917 ± 1.1	827.68227 ± 0.8	850.99744 ± 0.8	859.99471 ± 0.1	882.2126 ± 0.60
w	17.63777 ± 0.08	20.9764 ± 5.088	17.27971 ± 5.78	16.45765 ± 2.01	15.78736 ± 2.54	10.70084 ± 0.40	3.59959 ± 1.822
A	6346.09259 ± 23	405.22989 ± 317	1147.00318 ± 82	1455.75433 ± 55	675.86869 ± 182	1640.2288 ± 127	25.55584 ± 9.77
Reduced	3.27458						
R-Square (	0.99844						
Adj. R-Squ	0.99834						

Figure S15: Raman spectrum and fitting statistics of **KUPS-1** heated to 40 °C from **Figure 2** in the main text.

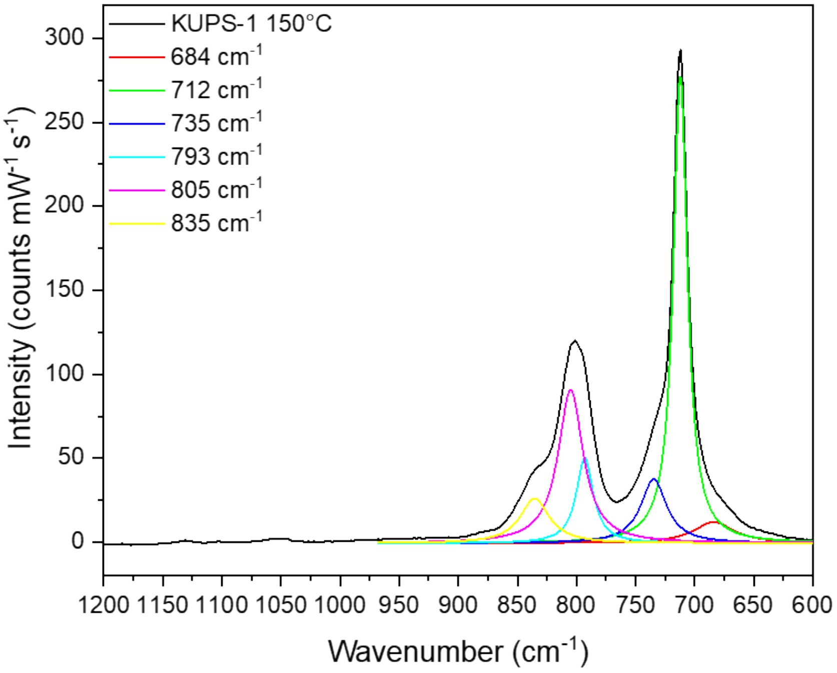

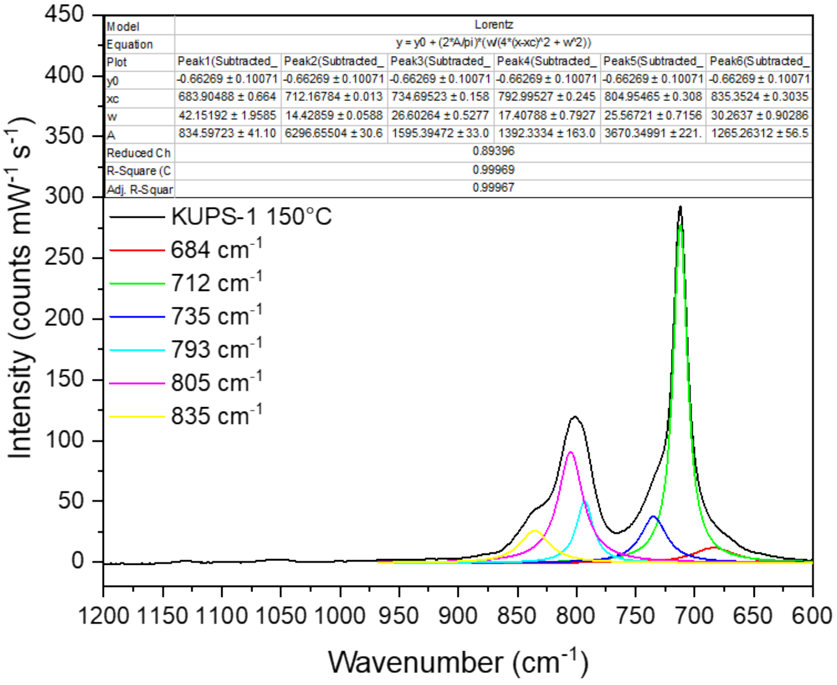

Model	Lorentz					
Equation	y = y0 + (2*A/pi)*(w/(4*(x-xc)^2 + w^2))					
Plot	Peak1(Subtracted_	Peak2(Subtracted_	Peak3(Subtracted_	Peak4(Subtracted_	Peak5(Subtracted_	Peak6(Subtracted_
y0	-0.66269 ± 0.10071	-0.66269 ± 0.10071	-0.66269 ± 0.10071	-0.66269 ± 0.10071	-0.66269 ± 0.10071	-0.66269 ± 0.10071
xc	683.90488 ± 0.664	712.16784 ± 0.013	734.69523 ± 0.158	792.99527 ± 0.245	804.95465 ± 0.308	835.3524 ± 0.3035
w	42.15192 ± 1.9585	14.42859 ± 0.0588	26.60264 ± 0.5277	17.40788 ± 0.7927	25.56721 ± 0.7156	30.2637 ± 0.90286
A	834.59723 ± 41.10	6296.65504 ± 30.6	1595.39472 ± 33.0	1392.3334 ± 163.0	3670.34991 ± 221.	1265.26312 ± 56.5
Reduced Ch	0.89396					
R-Square (C	0.99969					
Adj. R-Squar	0.99967					

Figure S16: Raman spectrum and fitting statistics of **KUPS-1** heated to 150 °C from **Figure 2** in the main text.

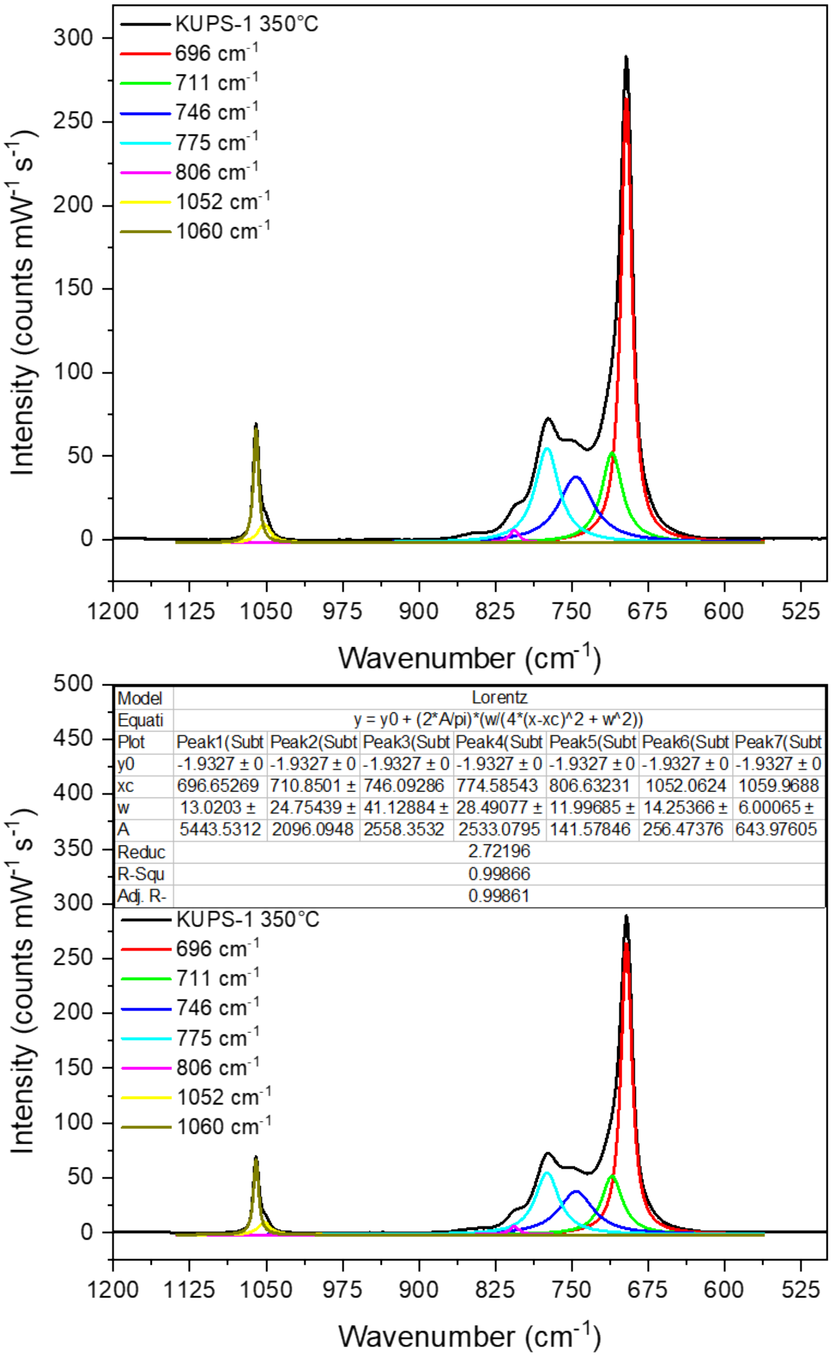

Model	Lorentz						
Equati	y = y0 + (2*A/pi)*(w/(4*(x-xc)^2 + w^2))						
Plot	Peak1(Subt	Peak2(Subt	Peak3(Subt	Peak4(Subt	Peak5(Subt	Peak6(Subt	Peak7(Subt
y0	-1.9327 ± 0	-1.9327 ± 0	-1.9327 ± 0	-1.9327 ± 0	-1.9327 ± 0	-1.9327 ± 0	-1.9327 ± 0
xc	696.65269	710.8501 ±	746.09286	774.58543	806.63231	1052.0624	1059.9688
w	13.0203 ±	24.75439 ±	41.12884 ±	28.49077 ±	11.99685 ±	14.25366 ±	6.00065 ±
A	5443.5312	2096.0948	2558.3532	2533.0795	141.57846	256.47376	643.97605
Reduc	2.72196						
R-Squ	0.99866						
Adj. R-	0.99861						

Figure S17: Raman spectrum and fitting statistics of **KUPS-1** heated to 350 °C from **Figure 2** in the main text.

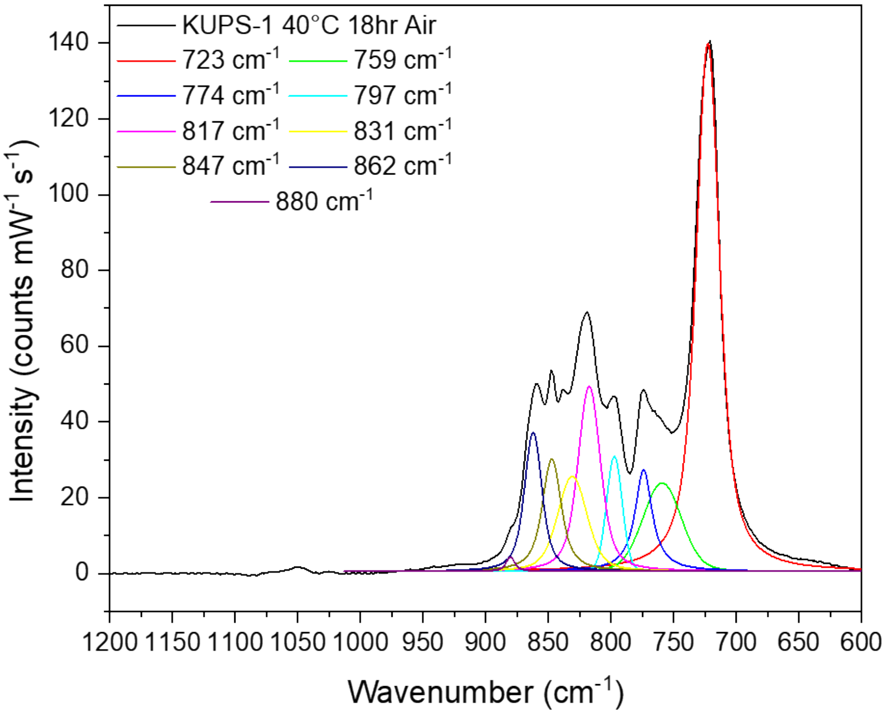

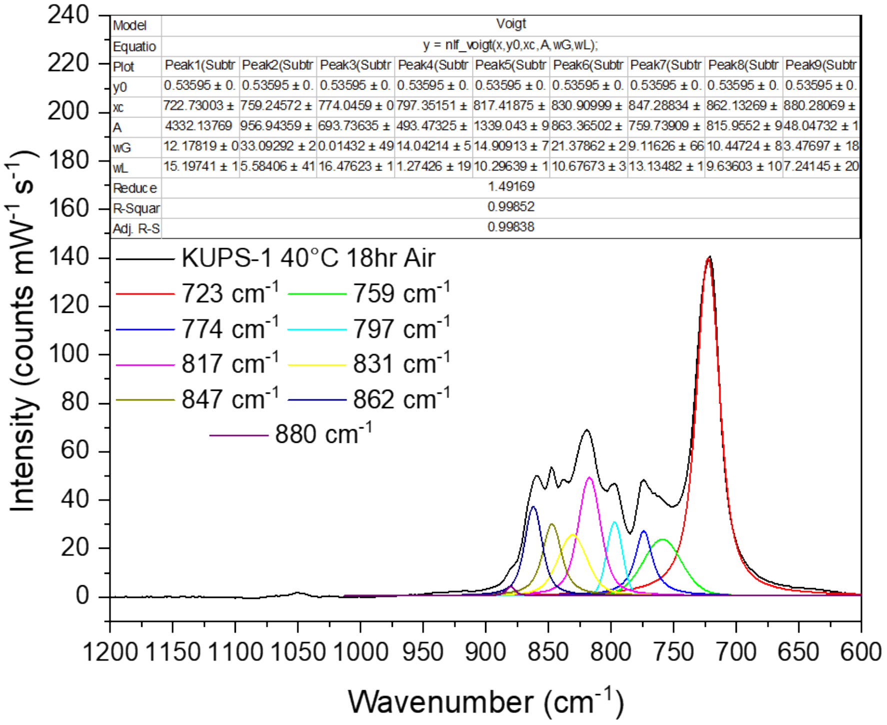

Model	Voigt								
Equatio	y = nlf_voigt(x,y0,xc,A,wG,wL);								
Plot	Peak1(Subtr	Peak2(Subtr	Peak3(Subtr	Peak4(Subtr	Peak5(Subtr	Peak6(Subtr	Peak7(Subtr	Peak8(Subtr	Peak9(Subtr
y0	0.53595 ± 0.	0.53595 ± 0.	0.53595 ± 0.	0.53595 ± 0.	0.53595 ± 0.	0.53595 ± 0.	0.53595 ± 0.	0.53595 ± 0.	0.53595 ± 0.
xc	722.73003 ±	759.24572 ±	774.0459 ± 0	797.35151 ±	817.41875 ±	830.90999 ±	847.28834 ±	862.13269 ±	880.28069 ±
A	4332.13769	956.94359 ±	693.73635 ±	493.47325 ±	1339.043 ± 9	863.36502 ±	759.73909 ±	815.9552 ± 9	48.04732 ± 1
wG	12.17819 ± 0	33.09292 ± 2	0.01432 ± 49	14.04214 ± 5	14.90913 ± 7	21.37862 ± 2	9.11626 ± 66	10.44724 ± 8	3.47697 ± 18
wL	15.19741 ± 1	5.58406 ± 41	16.47623 ± 1	1.27426 ± 19	10.29639 ± 1	10.67673 ± 3	13.13482 ± 1	9.63603 ± 10	7.24145 ± 20
Reduce	1.49169								
R-Squar	0.99852								
Adj. R-S	0.99838								

Figure S18: Raman spectrum and fitting statistics of **KUPS-1** heated isothermally at 40 °C for 18 hours in open air (**Figure 4A** in the main text).

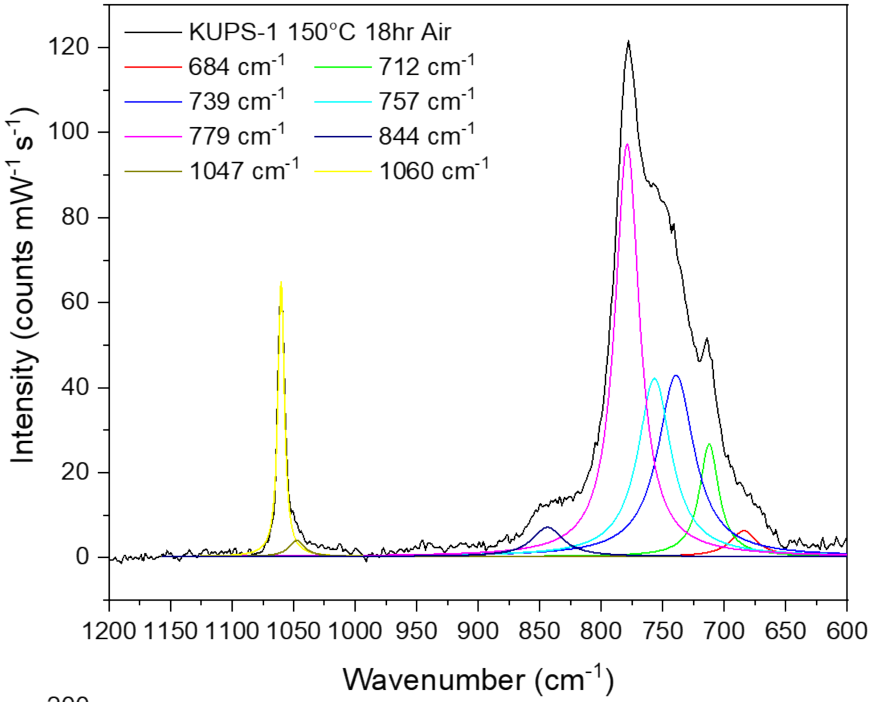

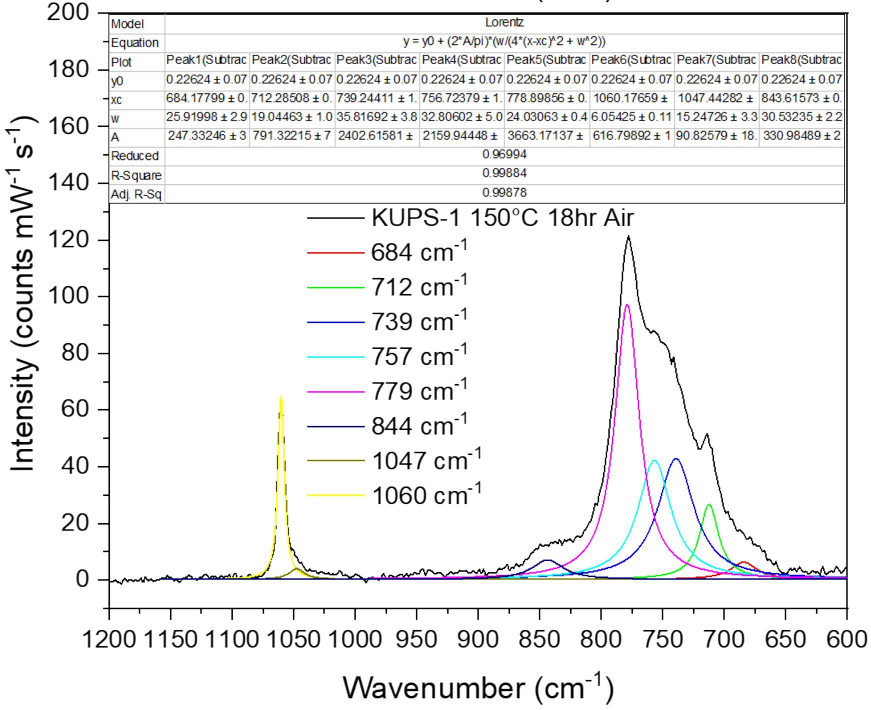

Model	Lorentz							
Equation	y = y0 + (2*A/pi)*(w/(4*(x-xc)^2 + w^2))							
Plot	Peak1(Subtrac	Peak2(Subtrac	Peak3(Subtrac	Peak4(Subtrac	Peak5(Subtrac	Peak6(Subtrac	Peak7(Subtrac	Peak8(Subtrac
y0	0.22624 ± 0.07	0.22624 ± 0.07	0.22624 ± 0.07	0.22624 ± 0.07	0.22624 ± 0.07	0.22624 ± 0.07	0.22624 ± 0.07	0.22624 ± 0.07
xc	684.17799 ± 0.	712.28508 ± 0.	739.24411 ± 1.	756.72379 ± 1.	778.89856 ± 0.	1060.17659 ±	1047.44282 ±	843.61573 ± 0.
w	25.91998 ± 2.9	19.04463 ± 1.0	35.81692 ± 3.8	32.80602 ± 5.0	24.03063 ± 0.4	6.05425 ± 0.11	15.24726 ± 3.3	30.53235 ± 2.2
A	247.33246 ± 3	791.32215 ± 7	2402.61581 ±	2159.94448 ±	3663.17137 ±	616.79892 ± 1	90.82579 ± 18.	330.98489 ± 2
Reduced	0.96994							
R-Square	0.99884							
Adj. R-Sq	0.99878							

Figure S19: Raman spectrum and fitting statistics of **KUPS-1** heated isothermally at 150 °C for 18 hours in open air (**Figure 4B** in the main text).

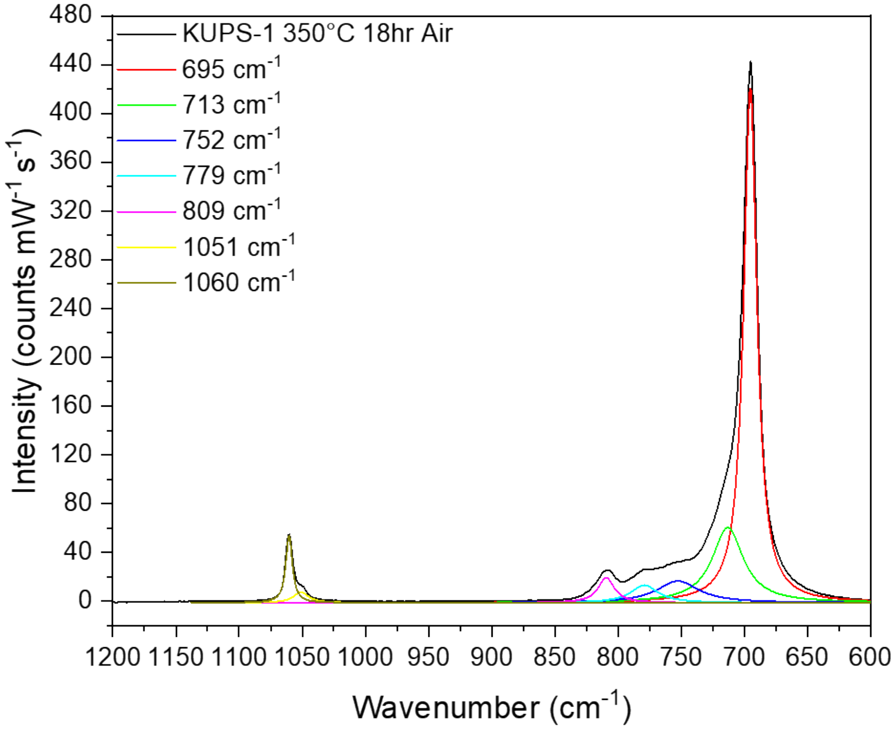

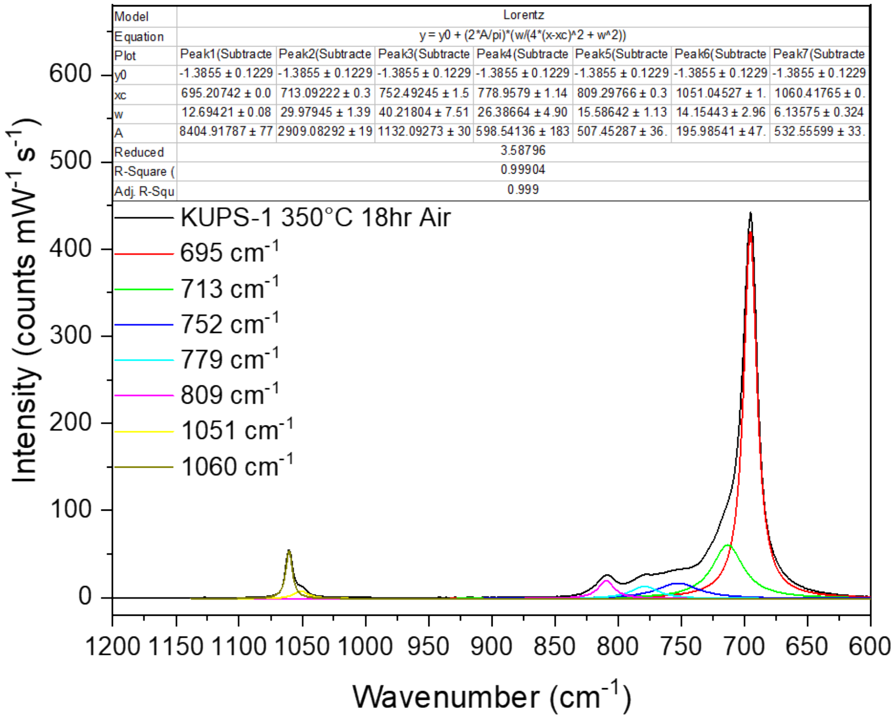

Model	Lorentz						
Equation	y = y0 + (2*A/pi)*(w/(4*(x-xc)^2 + w^2))						
Plot	Peak1(Subtracte	Peak2(Subtracte	Peak3(Subtracte	Peak4(Subtracte	Peak5(Subtracte	Peak6(Subtracte	Peak7(Subtracte
y0	-1.3855 ± 0.1229	-1.3855 ± 0.1229	-1.3855 ± 0.1229	-1.3855 ± 0.1229	-1.3855 ± 0.1229	-1.3855 ± 0.1229	-1.3855 ± 0.1229
xc	695.20742 ± 0.0	713.09222 ± 0.3	752.49245 ± 1.5	778.9579 ± 1.14	809.29766 ± 0.3	1051.04527 ± 1.	1060.41765 ± 0.
w	12.69421 ± 0.08	29.97945 ± 1.39	40.21804 ± 7.51	26.38664 ± 4.90	15.58642 ± 1.13	14.15443 ± 2.96	6.13575 ± 0.324
A	8404.91787 ± 77	2909.08292 ± 19	1132.09273 ± 30	598.54136 ± 183	507.45287 ± 36.	195.98541 ± 47.	532.55599 ± 33.
Reduced	3.58796						
R-Square (	0.99904						
Adj. R-Squ	0.999						

Figure S20: Raman spectrum and fitting statistics of **KUPS-1** heated isothermally at 350 °C for 18 hours in open air (**Figure 4C** in the main text)

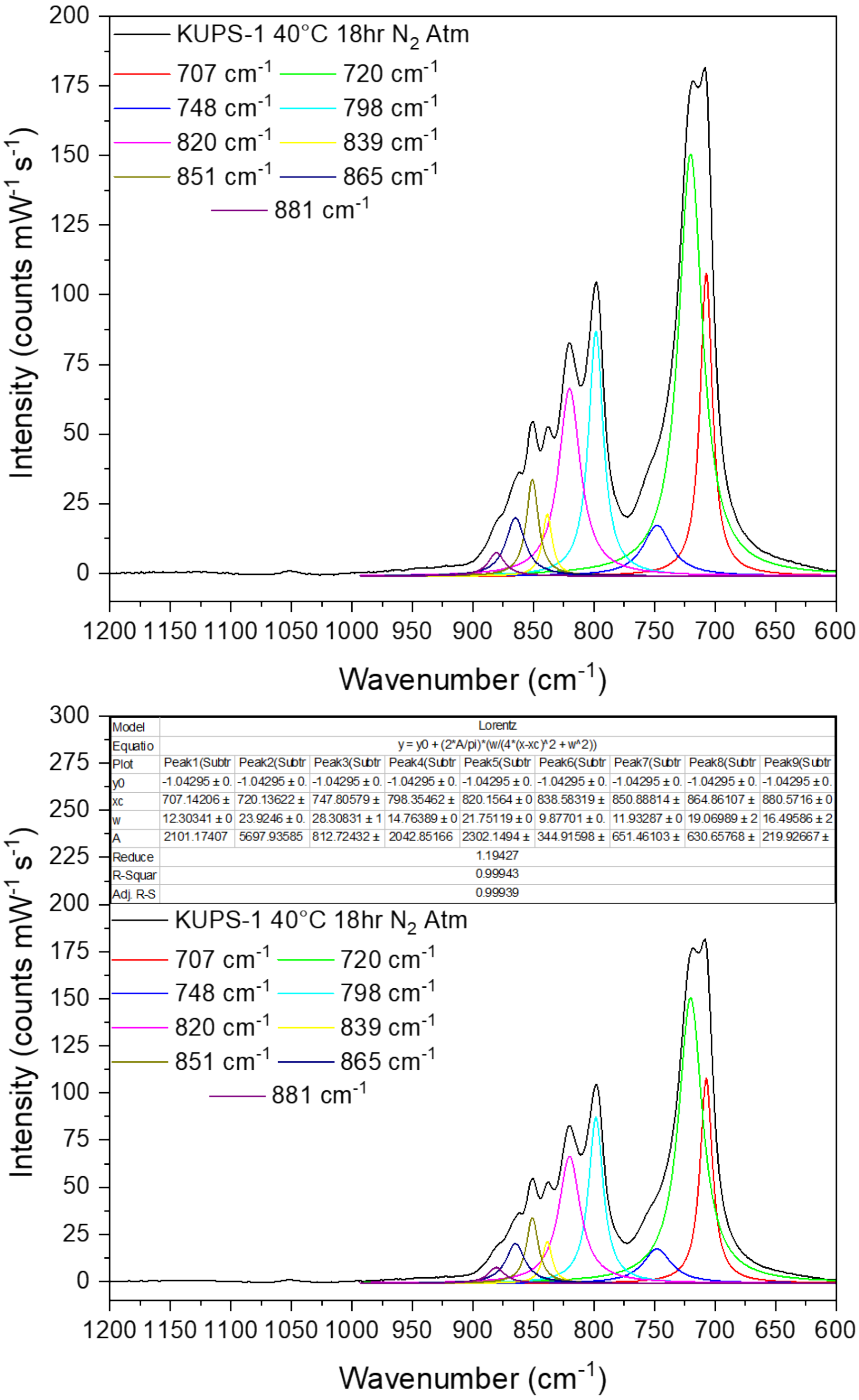

Model	Lorentz								
Equatio	y = y0 + (2*A/pi)*(w/(4*(x-xc)^2 + w^2))								
Plot	Peak1(Subtr	Peak2(Subtr	Peak3(Subtr	Peak4(Subtr	Peak5(Subtr	Peak6(Subtr	Peak7(Subtr	Peak8(Subtr	Peak9(Subtr
y0	-1.04295 ± 0.	-1.04295 ± 0.	-1.04295 ± 0.	-1.04295 ± 0.	-1.04295 ± 0.	-1.04295 ± 0.	-1.04295 ± 0.	-1.04295 ± 0.	-1.04295 ± 0.
xc	707.14206 ±	720.13622 ±	747.80579 ±	798.35462 ±	820.1564 ± 0	838.58319 ±	850.88814 ±	864.86107 ±	880.5716 ± 0
w	12.30341 ± 0	23.9246 ± 0.	28.30831 ± 1	14.76389 ± 0	21.75119 ± 0	9.87701 ± 0.	11.93287 ± 0	19.06989 ± 2	16.49586 ± 2
A	2101.17407	5697.93585	812.72432 ±	2042.85166	2302.1494 ±	344.91598 ±	651.46103 ±	630.65768 ±	219.92667 ±
Reduce	1.19427								
R-Squar	0.99943								
Adj. R-S	0.99939								

Figure S21: Raman spectrum and fitting statistics of **KUPS-1** heated isothermally at 40 °C for 18 hours under N_2 atmosphere (**Figure 4D** in the main text).

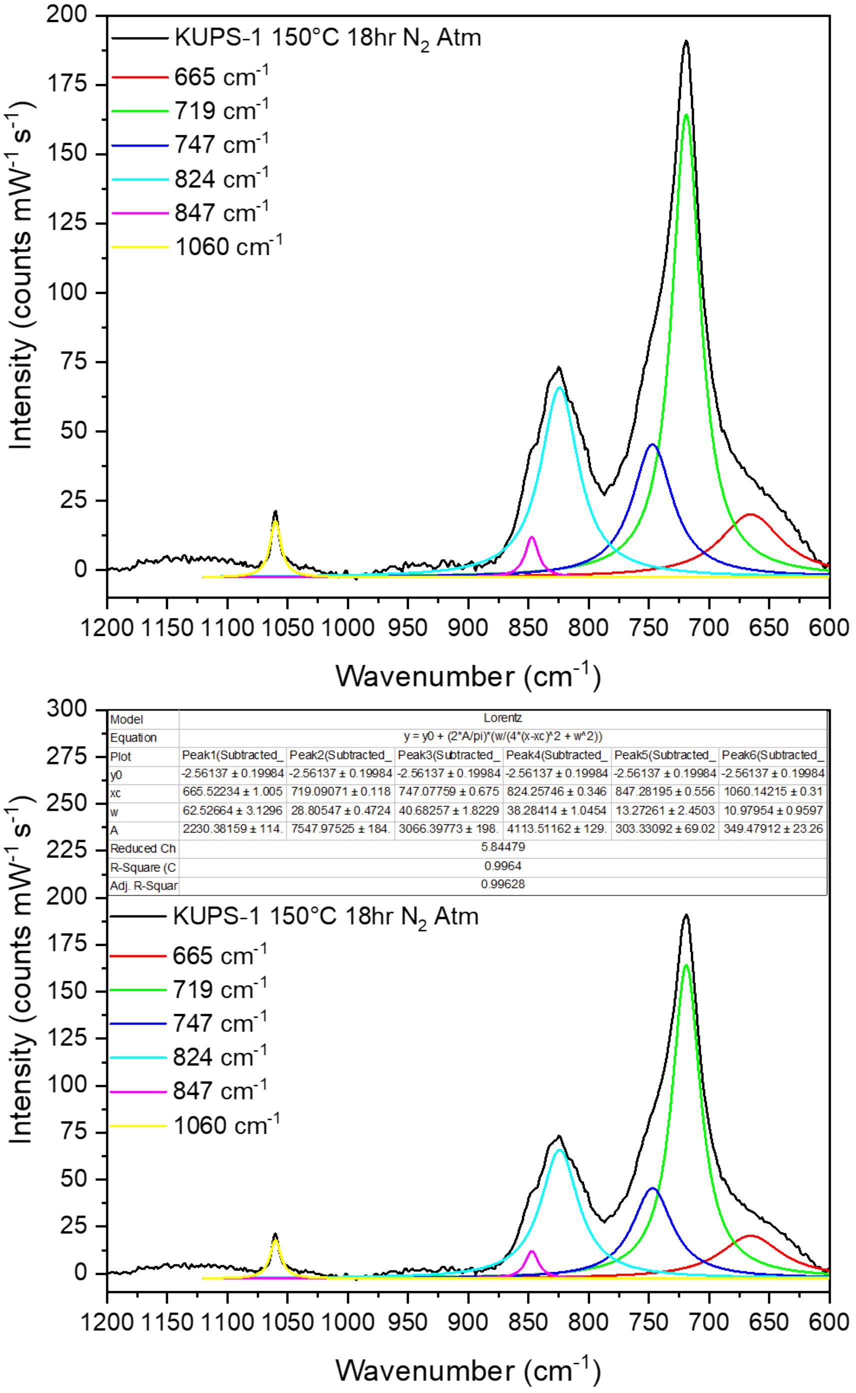

Model	Lorentz					
Equation	y = y0 + (2*A/pi)*(w/(4*(x-xc)^2 + w^2))					
Plot	Peak1(Subtracted_	Peak2(Subtracted_	Peak3(Subtracted_	Peak4(Subtracted_	Peak5(Subtracted_	Peak6(Subtracted_
y0	-2.56137 ± 0.19984	-2.56137 ± 0.19984	-2.56137 ± 0.19984	-2.56137 ± 0.19984	-2.56137 ± 0.19984	-2.56137 ± 0.19984
xc	665.52234 ± 1.005	719.09071 ± 0.118	747.07759 ± 0.675	824.25746 ± 0.346	847.28195 ± 0.556	1060.14215 ± 0.31
w	62.52664 ± 3.1296	28.80547 ± 0.4724	40.68257 ± 1.8229	38.28414 ± 1.0454	13.27261 ± 2.4503	10.97954 ± 0.9597
A	2230.38159 ± 114.	7547.97525 ± 184.	3066.39773 ± 198.	4113.51162 ± 129.	303.33092 ± 69.02	349.47912 ± 23.26
Reduced Ch	5.84479					
R-Square (C	0.9964					
Adj. R-Squar	0.99628					

Figure S22: Raman spectrum and fitting statistics of **KUPS-1** heated isothermally at 150 °C for 18 hours under N_2 atmosphere (**Figure 4E** in the main text).

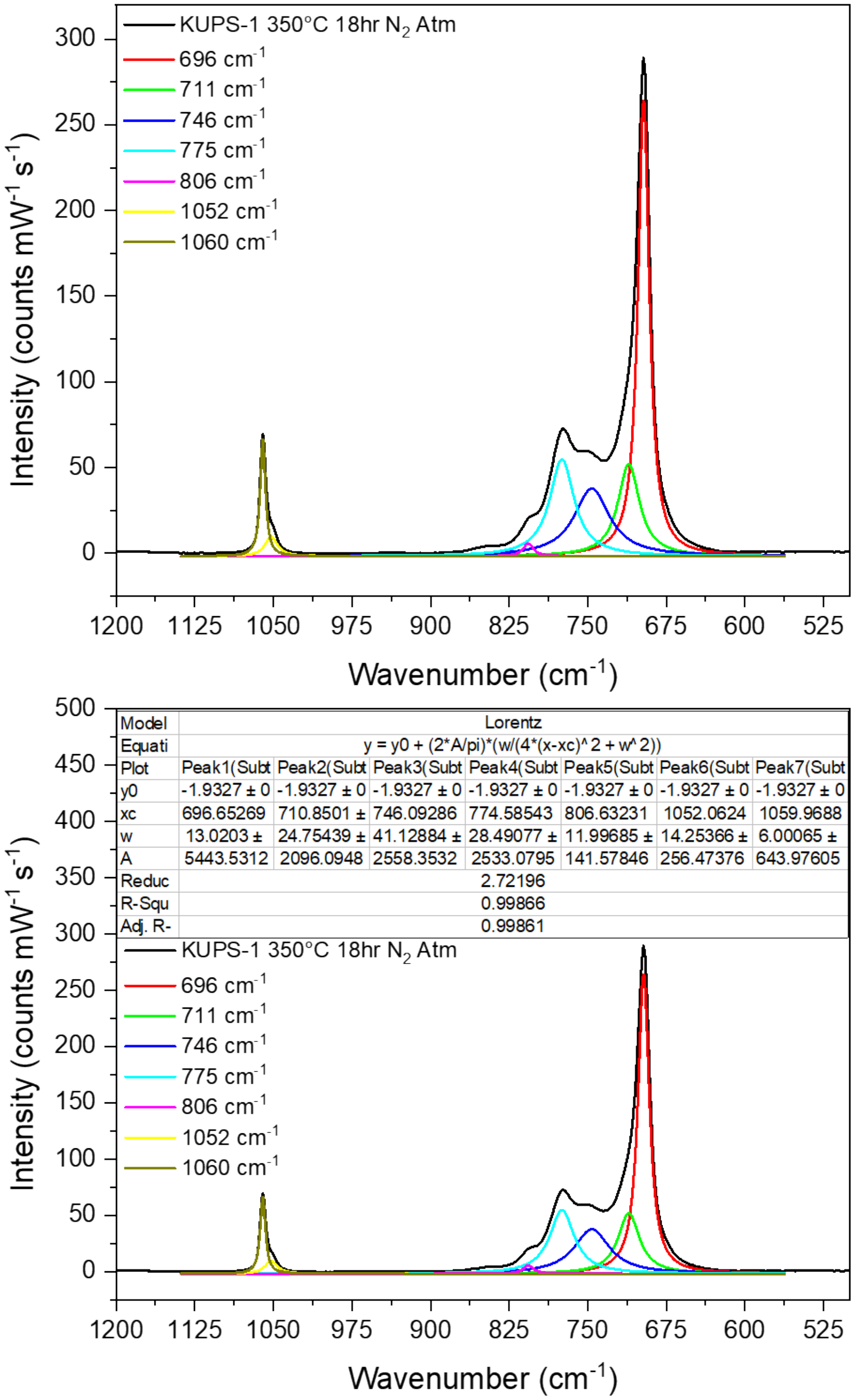

Model	Lorentz						
Equati	y = y0 + (2*A/pi)*(w/(4*(x-xc)^2 + w^2))						
Plot	Peak1(Subt	Peak2(Subt	Peak3(Subt	Peak4(Subt	Peak5(Subt	Peak6(Subt	Peak7(Subt
y0	-1.9327 ± 0	-1.9327 ± 0	-1.9327 ± 0	-1.9327 ± 0	-1.9327 ± 0	-1.9327 ± 0	-1.9327 ± 0
xc	696.65269	710.8501 ±	746.09286	774.58543	806.63231	1052.0624	1059.9688
w	13.0203 ±	24.75439 ±	41.12884 ±	28.49077 ±	11.99685 ±	14.25366 ±	6.00065 ±
A	5443.5312	2096.0948	2558.3532	2533.0795	141.57846	256.47376	643.97605
Reduc	2.72196						
R-Squ	0.99866						
Adj. R-	0.99861						

Figure S23: Raman spectrum and fitting statistics of **KUPS-1** heated isothermally at 350 °C for 18 hours under N_2 atmosphere (**Figure 4F** in the main text).

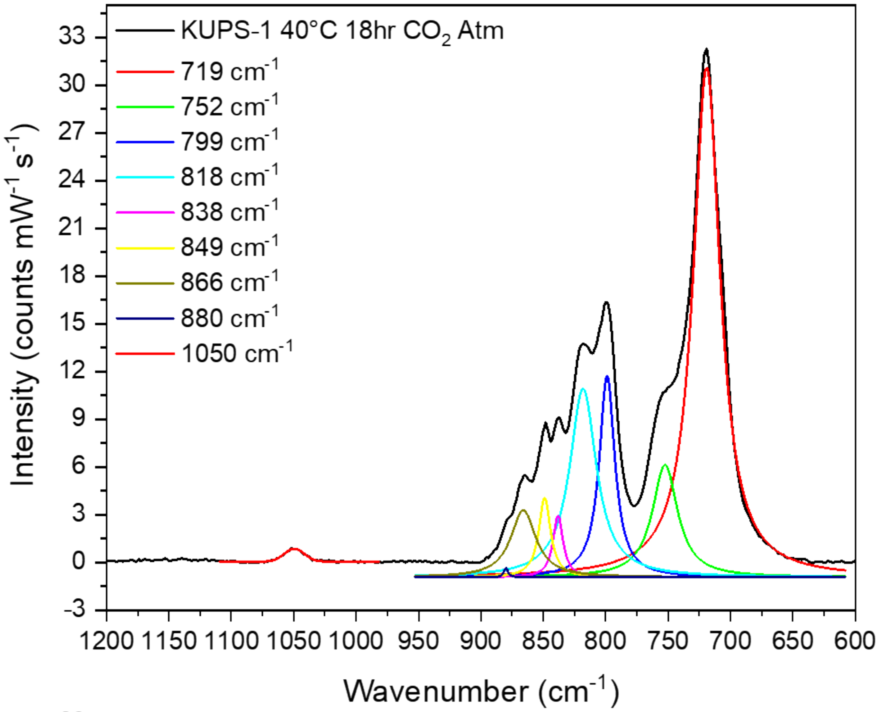

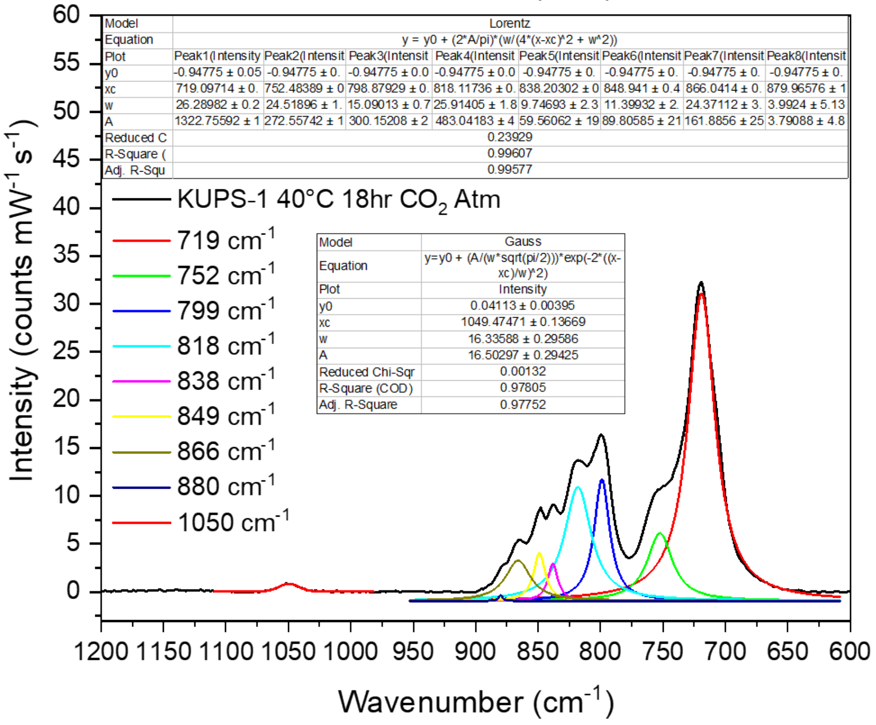

Figure S24: Raman spectrum and fitting statistics of **KUPS-1** heated isothermally at 40 °C for 18 hours under CO_2 atmosphere (**Figure 4G** in the main text).

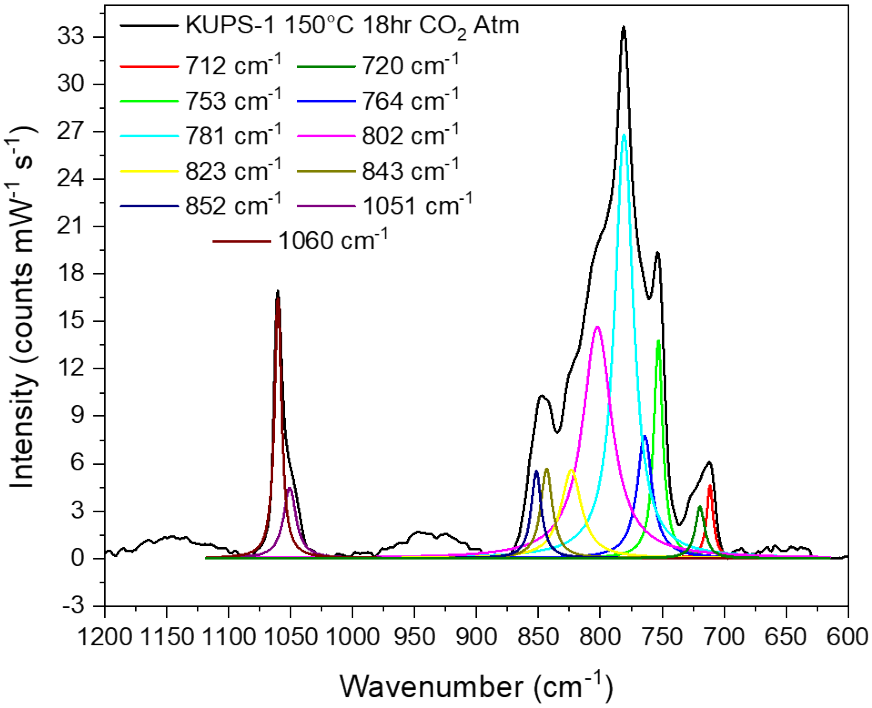

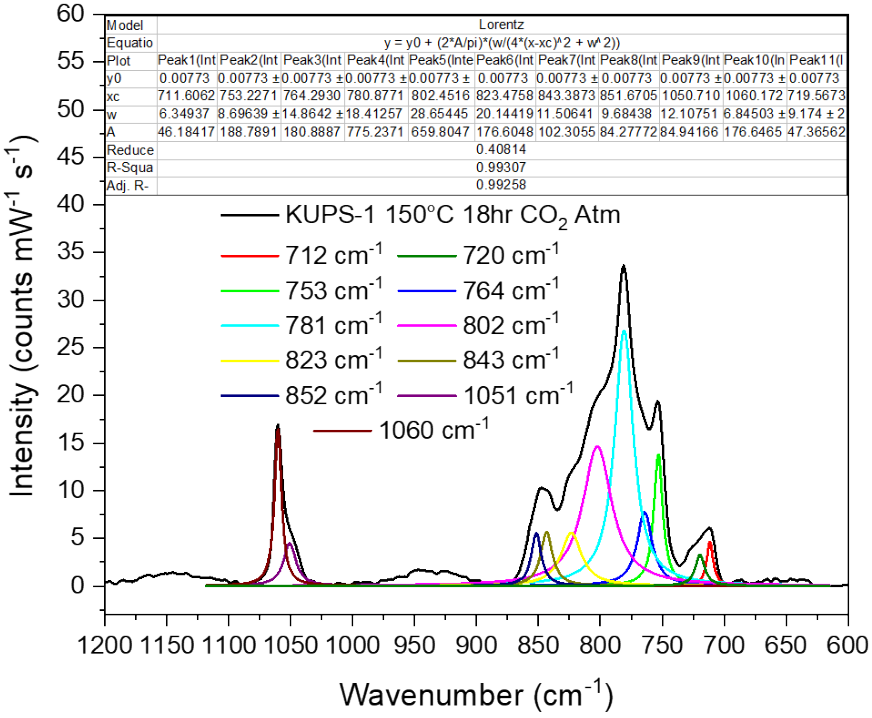

Model	Lorentz										
Equatio	y = y0 + (2*A/pi)*(w/(4*(x-xc)^2 + w^2))										
Plot	Peak1(Int	Peak2(Int	Peak3(Int	Peak4(Int	Peak5(Inte	Peak6(Int	Peak7(Int	Peak8(Int	Peak9(Int	Peak10(In	Peak11(I
y0	0.00773	0.00773 ±	0.00773 ±	0.00773 ±	0.00773 ±	0.00773	0.00773 ±	0.00773	0.00773 ±	0.00773 ±	0.00773
xc	711.6062	753.2271	764.2930	780.8771	802.4516	823.4758	843.3873	851.6705	1050.710	1060.172	719.5673
w	6.34937	8.69639 ±	14.8642 ±	18.41257	28.65445	20.14419	11.50641	9.68438	12.10751	6.84503 ±	9.174 ± 2
A	46.18417	188.7891	180.8887	775.2371	659.8047	176.6048	102.3055	84.27772	84.94166	176.6465	47.36562
Reduce	0.40814										
R-Squa	0.99307										
Adj. R-	0.99258										

Figure S25: Raman spectrum and fitting statistics of **KUPS-1** heated isothermally at 150 °C for 18 hours under CO_2 atmosphere (**Figure 4H** in the main text).

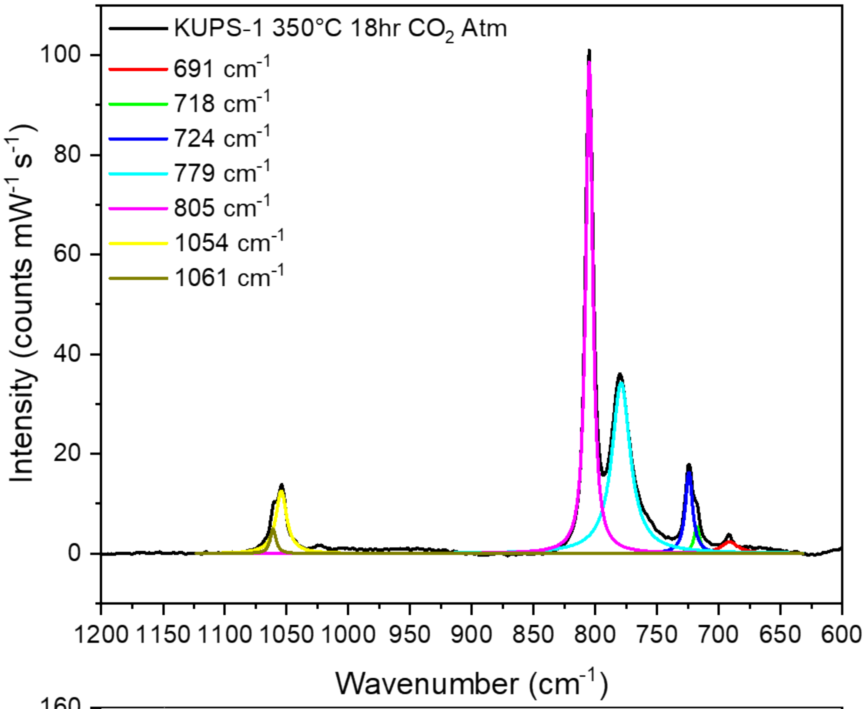

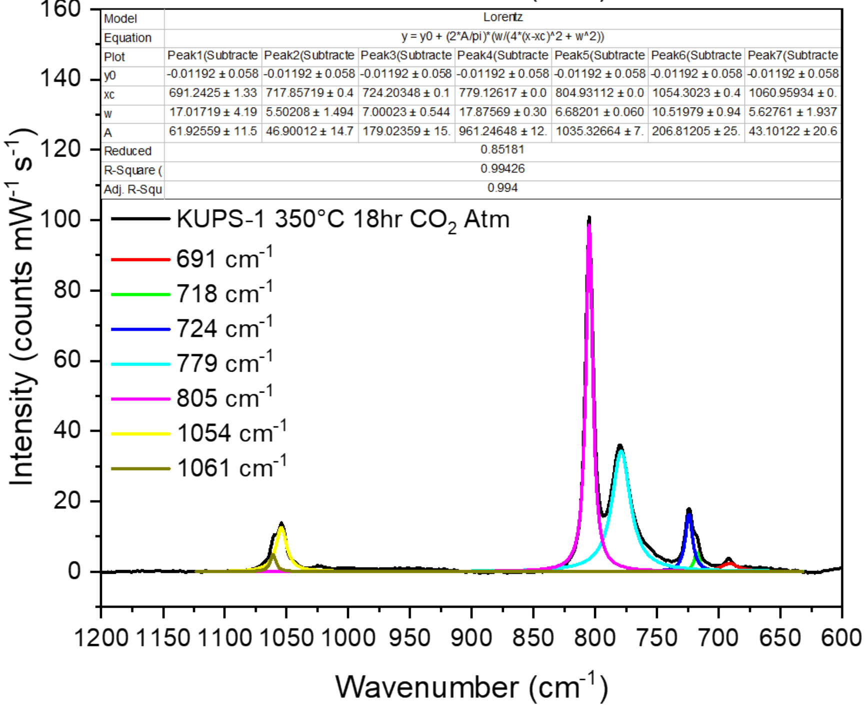

Model	Lorentz						
Equation	y = y0 + (2*A/pi)*(w/(4*(x-xc)^2 + w^2))						
Plot	Peak1(Subtracte	Peak2(Subtracte	Peak3(Subtracte	Peak4(Subtracte	Peak5(Subtracte	Peak6(Subtracte	Peak7(Subtracte
y0	-0.01192 ± 0.058	-0.01192 ± 0.058	-0.01192 ± 0.058	-0.01192 ± 0.058	-0.01192 ± 0.058	-0.01192 ± 0.058	-0.01192 ± 0.058
xc	691.2425 ± 1.33	717.85719 ± 0.4	724.20348 ± 0.1	779.12617 ± 0.0	804.93112 ± 0.0	1054.3023 ± 0.4	1060.95934 ± 0.
w	17.01719 ± 4.19	5.50208 ± 1.494	7.00023 ± 0.544	17.87569 ± 0.30	6.68201 ± 0.060	10.51979 ± 0.94	5.62761 ± 1.937
A	61.92559 ± 11.5	46.90012 ± 14.7	179.02359 ± 15.	961.24648 ± 12.	1035.32664 ± 7.	206.81205 ± 25.	43.10122 ± 20.6
Reduced	0.85181						
R-Square (	0.99426						
Adj. R-Squ	0.994						

Figure S26: Raman spectrum and fitting statistics of **KUPS-1** heated isothermally at 350 °C for 18 hours under CO_2 atmosphere (**Figure 4I** in the main text).

Thermodynamics Calculations

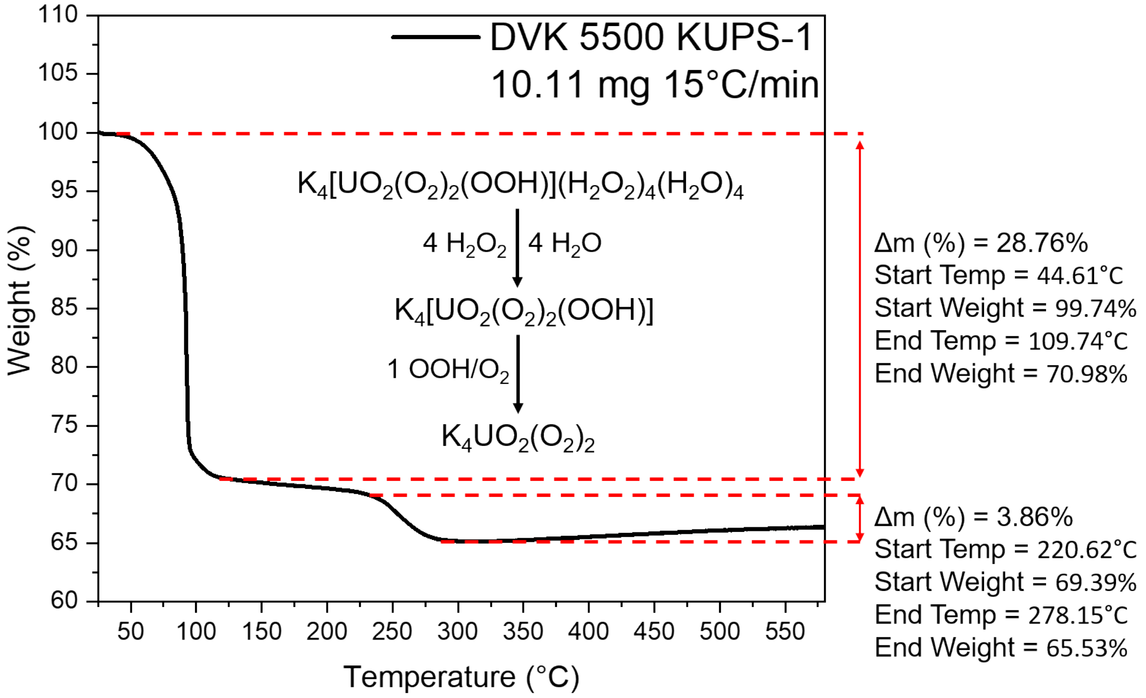

Figure S27: Thermogravimetric analysis curve of **KUPS-1** with indicated weight losses.

Theoretical Weight Loss

$$\frac{4 * Mr(H_2O) + 4 * Mr(H_2O_2)}{Mr(KUPS-1)} * 100\% = \frac{4 * 18\frac{gr}{mole} + 4 * 34\frac{gr}{mole}}{731.5\,\frac{gr}{mole}} * 100\% = 28.43\%$$

$$\frac{1 * Mr(O_2)}{Mr(KUPS-1)} * 100\% = \frac{1 * 32\frac{gr}{mole}}{731.5\,\frac{gr}{mole}} * 100\% = 4.37\%$$

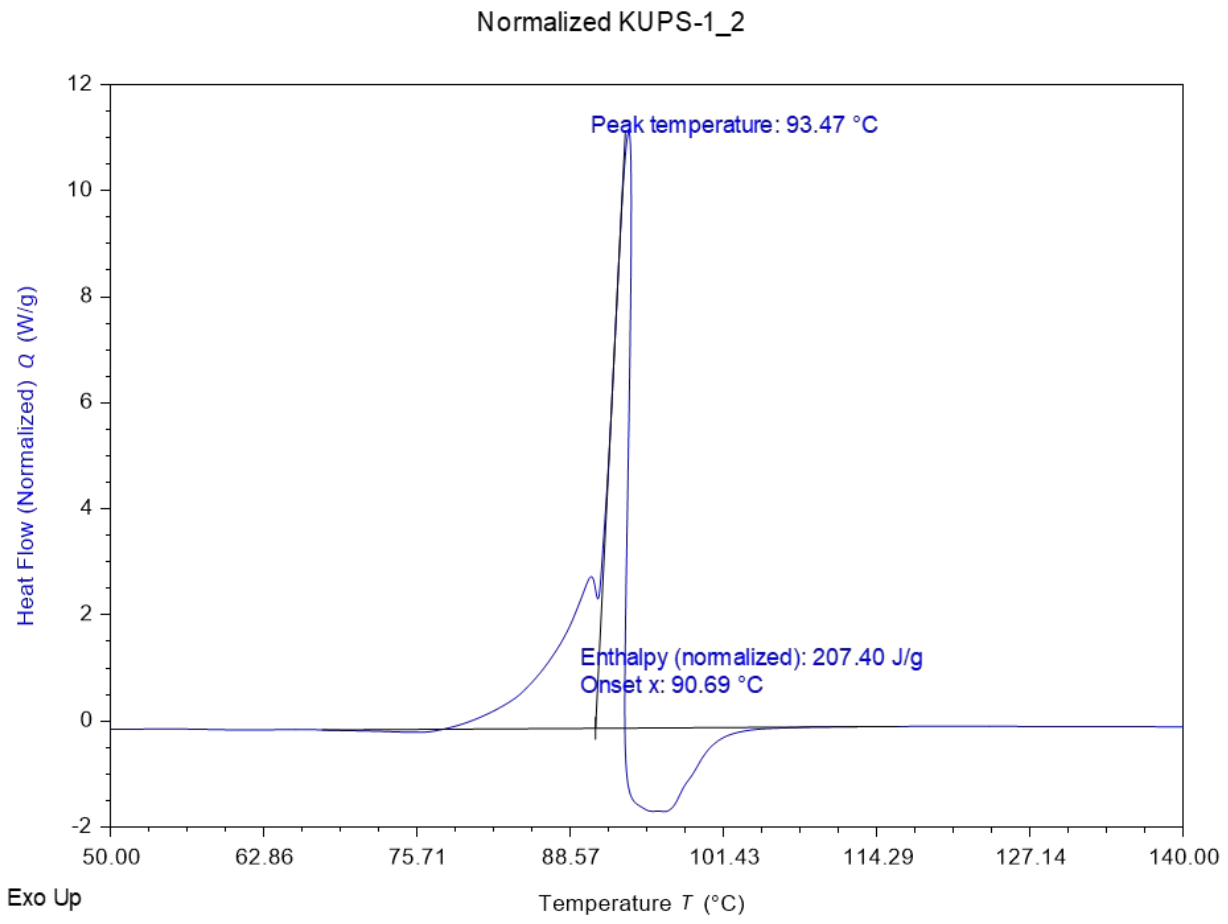

Figure S28: DSC curve of **KUPS-1** in the temperature range from 50 °C to 140 °C processed in TRIOS software.

$$207.40\frac{J}{gr} * 730.5\frac{gr}{mole} = 151{,}505\,\frac{J}{mole} = 151.5\,\frac{kJ}{mole}$$

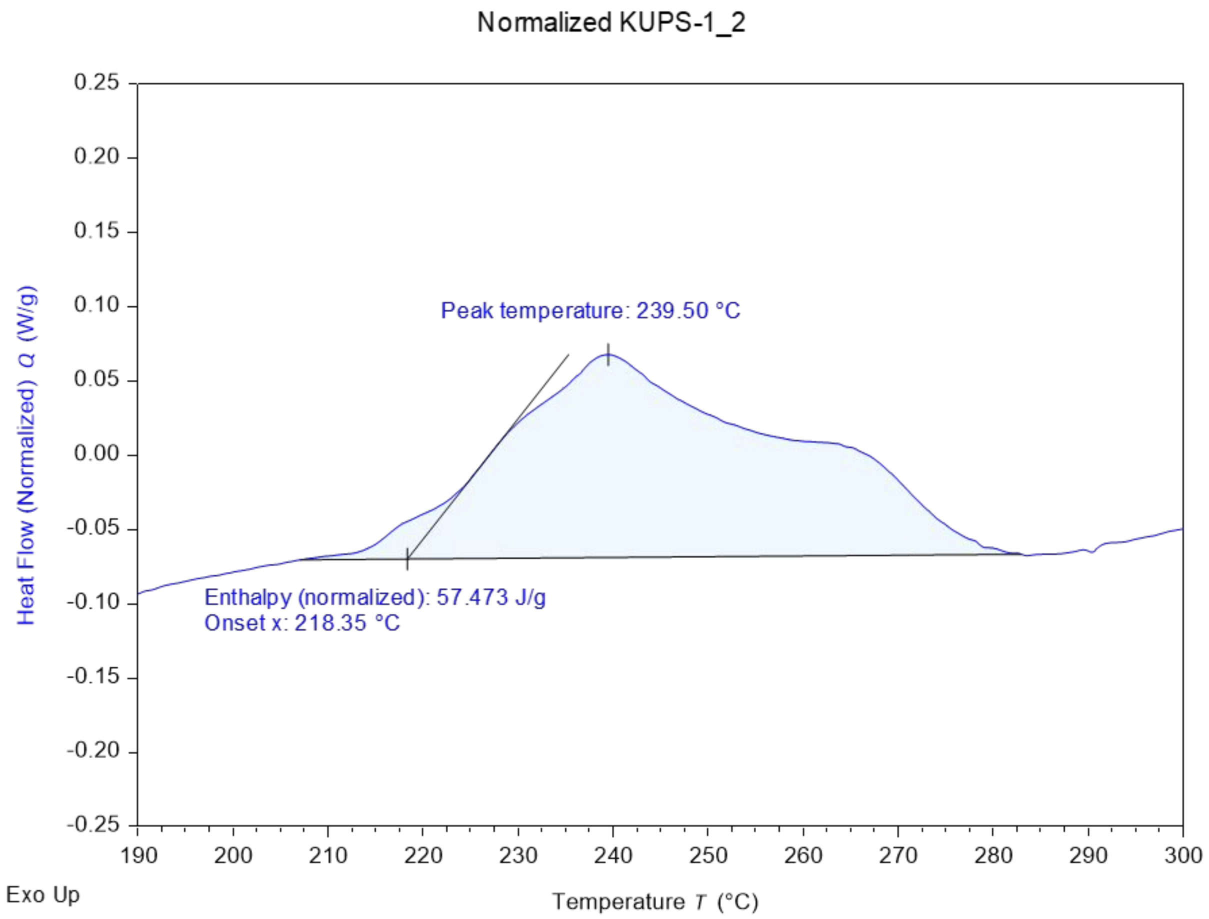

Figure S29: DSC curve of **KUPS-1** in the temperature range from 190 °C to 300 °C processed in TRIOS software.

$$57.473\frac{J}{gr} * 522.42\frac{gr}{mole} = 30{,}025\,\frac{J}{mole} = 30.02\,\frac{kJ}{mole}$$

Table 1. Calculation of the average enthalpy for the exothermic transition at 95 °C for **KUPS-1** material.

Experimental Enthalpy Values (kJ/mole)	*Mean*	*Standard Deviation*	*Confidence Interval at 95%*
-151.50			
-150.38	-148.78	3.77	4.269
-144.48			

Table 2. Calculation of the average enthalpy for the exothermic transition at 240 °C for **KUPS-1** material.

Experimental Enthalpy Values (kJ/mole)	*Mean*	*Standard Deviation*	*Confidence Interval at 95%*
-31.125			
-30.025	-29.046	1.588	1.556
-27.533			
